# Osteoking promotes bone formation and bone defect repair through ZBP1–STAT1–PKR–MLKL-mediated necroptosis

**DOI:** 10.1186/s13020-024-00883-4

**Published:** 2024-01-18

**Authors:** Suya Zhang, Yudong Liu, Zhaochen Ma, Shuangrong Gao, Lin Chen, Honggang Zhong, Chu Zhang, Tao Li, Weiheng Chen, Yanqiong Zhang, Na Lin

**Affiliations:** 1 Science and Technology Innovation Center, Guangzhou University of Chinese Medicine, 12 Airport Road, Baiyun District, Guangzhou, 510405 China; 2 Institute of Chinese Materia Medica, China Academy of Chinese Medical Sciences, No. 16, Nanxiaojie, Dongzhimennei, Beijing, 100700 China; 3 BioMechanics Lab, Wang Jing Hospital, China Academy of Chinese Medical Sciences, Beijing, 100010 China; 4 Third Affiliated Hospital of Beijing University of Chinese Medicine, No. 51 Anwai Xiaoguanjie, Chaoyang District, Beijing, 100029 China

**Keywords:** Bone defect repair, Bone formation, Osteoking, Necroptosis, Transcriptome-based network investigation

## Abstract

**Background:**

Osteoking has been used for fracture therapy with a satisfying clinical efficacy. However, its therapeutic properties and the underlying mechanisms remain elusive.

**Method:**

A bone defect rat model was established to evaluate the pharmacological effects of Osteoking by the dynamic observation of X-ray, micro-CT and histopathologic examination. Transcriptome profiling was performed to identify bone defect-related genes and Osteoking effective targets. Then, a “disease-related gene–drug target” interaction network was constructed and a list of key network targets were screened, which were experimentally verified.

**Results:**

Osteoking effectively promoted bone defect repair in rats by accelerating the repair of cortical bone and the growth of trabeculae. Histopathologically, the bone defect rats displayed lower histopathologic scores in cortical bone, cancellous bone and bone connection than normal controls. In contrast, Osteoking exerted a favorable effect with a dose-dependent manner. The abnormal serum levels of bone turnover markers, bone growth factors and bone metabolism-related biochemical indexes in bone defect rats were also reversed by Osteoking treatment. Following the transcriptome-based network investigation, we hypothesized that osteoking might attenuate the levels of ZBP1–STAT1–PKR–MLKL-mediated necroptosis involved into bone defect. Experimentally, the expression levels of ZBP1, STAT1, PKR and the hallmark inflammatory cytokines for the end of necroptosis were distinctly elevated in bone defect rats, but were all effectively reversed by Osteoking treatment, which were also suppressed the activities of RIPK1, RIPK3 and MLKL in bone tissue supernatants.

**Conclusions:**

Osteoking may promote bone formation and bone defect repair by regulating ZBP1–STAT1–PKR axis, leading to inhibit RIPK1/RIPK3/MLKL activation-mediated necroptosis.

**Supplementary Information:**

The online version contains supplementary material available at 10.1186/s13020-024-00883-4.

## Introduction

Fracture, resulting from external trauma, excessive pressure, or the surpassing of the bone’s strength threshold, may seriously impact patients’ physical functions and psychological well-being. It often leads to a substantial escalation in the risk of disability, financial burden and mortality. The prevalence of fractures is substantial, with an approximate mean of two fractures per individual throughout the course of their lifetime [1] and the mortality rate often exhibits its maximum levels within the initial year following a fracture. Notably, growing clinical evidence show that approximately 5–10% of fractures fail to heal and develop non-unions, and the mortality rate subsequent to a fracture are also increasing [2], underscoring the criticality of timely intervention. Fracture has been indicated to be influenced by various risk factors, with osteoporosis being a prominent contributor [3]. The existing therapeutics of fracture, including conservative and surgical approaches, may focus on facilitating the process of bone healing, regenerating mineralized tissue inside the fractured region and reinstating the mechanical integrity of the bone [1]. Regarding to pharmacotherapeutics, physicians may contemplate the utilization of bone density-regulating medications including bisphosphonates, selective estrogen receptor modulators, calcitonin, etc., to reduce the risk of fractures, and non-steroidal anti-inflammatory medicines and analgesics are also recommended to mitigate pain and discomfort [4]. However, due to the individual variability in response to existing therapeutics, the potential risks and side effects of inhibiting osteogenesis, and the high cost, more efficacious therapeutics are an ongoing concern.

Traditional Chinese medicine (TCM), as a kind of complementary and alternative medicine, have been used clinically for the treatment of various orthopedic diseases, particular for fracture [5–7]. TCM plays a crucial role in promoting bone formation and bone defect repair by using herbal prescriptions, acupuncture, diet therapy, massage, and exercise to maintain the state of equilibrium of the body [8]. Osteoking (known as Heng-Gu-Gu-Shang-Yu-He-Ji in Chinese) has been approved by Chinese State Food and Drug Administration and extensively used for the treatment of multiple orthopedic diseases, such as fracture, osteoporosis, osteoarthritis and osteonecrosis of the femoral head, with a satisfying clinical efficacy [9–12]. This prescription consists of nine Chinese herbs, including *Datura metel* L. (Yang Jin Hua), *Carthamus tinctorius* L. (Hong Hua), *Radix notoginseng* (San Qi), *Eucommia ulmoides* Oliv. (Du Zhong), *Radix ginseng* (Ren Shen), *Radix Astragali Mongolici* (Huang Qi), *Citrus reticulata Blanco* (Chen Pi), *Trionyx sinensis carapace* (Bie Jia) *and Schizophragma integrifolium* Oliv. (Zuan Di Feng). TCM theory holds that “the kidney governs the bone”, meaning that the wax and wane of kidney essence directly affects the growth, development, nutrition, and function of bones. Osteoking contains various Chinese herbs that exert effects of tonifying the kidneys, subsequently promote bone growth and repair. Accumulating pharmacological studies have revealed that this prescription may play a role in retarding cartilage degeneration, improving bone quality and enhancing calcium deposition [9, 11, 12]. However, the therapeutic properties and the underlying mechanisms of Osteoking for fracture therapy remain elusive, which may restrict the rational application of this prescription in clinical practice.

To address this issue, we herein aimed to evaluate the pharmacological effects and characteristics of Osteoking for fracture therapy based on a bone defect rat model and also investigate its underlying pharmacological mechanisms using an integrated strategy with transcriptome-based network investigation and experimental validation.

## Materials and methods

### Animals

Male Sprague–Dawley (SD) rats (*n* = 40, 200 ± 20 g in weight, 6–8 weeks old) were purchased from Beijing Vital River Laboratory Animal Technology Co., Ltd. (production license no: SCXK 2021-0006, Beijing, China). All rats were maintained in a room with a constant temperature of 24 ± 1 ℃ and with a 12-h light and dark cycle. The rats were allowed free access to water and food.

### Bone defect models

A bone defect rat model was established according to the description in previous studies [13–15]. Briefly, the rats were anesthetized via intraperitoneal injection of pentobarbital sodium solution (30 mg/kg) and were fixed on a rat surgical plate apparatus. A 1.5 cm longitudinal incision was made at the edge of the right middle tibia (where the tibial arc is most obvious). The surrounding muscles and blood vessels were carefully separated with blunt surgical instruments to fully expose the tibia. A 2.0-mm-diameter Kirschner pin was used as a drill bit to drill a completely penetrating circular hole in the mid-tibia. The surgery region was then flushed with 0.9% sodium chloride solution and sutured layer by layer. After the surgery, the rats were not restricted from walking freely and were injected intramuscularly with penicillin sodium solution for 3 consecutive days to avoid infection.

### Grouping and treatment

After the surgery, a total of 40 rats were randomly divided into five groups: normal control (*n* = 8), bone defect model (*n* = 8) and Osteoking treatment groups including Osteoking low-dose (1.3125 mL/kg, *n* = 8), Osteoking middle-dose (2.625 mL/kg, *n* = 8) and Osteoking high-dose (5.25 mL/kg, *n* = 8), which were equivalent to 0.5, 1, and 2 times the daily dosage for fracture patients in clinics, respectively. Both normal control and bone defect model groups were received the same volume of saline. All treatments were performed for 3 weeks via oral administration from the day after the surgery.

### Tissue collection and processing

On the 22nd day after the surgery, all rats were anesthetized by intraperitoneal injection of pentobarbital sodium (50 mg/kg). Blood samples were collected by the abdominal aortic method. Then, Trizol reagent was added to a portion of the blood sample and immediately transferred to liquid nitrogen for RNA sequencing (RNA-seq) detection, and the remaining blood sample was left at room temperature for 1 h. After that, it was centrifuged at 4 °C and 3000 rpm for 15 min, and the serum was collected and stored at − 20 °C for ELISA. The right tibia tissues collected from rats were used for three assays as following: one for microarray detection was immediately frozen in liquid nitrogen; one for micro-CT analysis and histopathological observation was fixed in 10% buffered formalin; and the remaining part was stored at − 80 °C for western blotting and ELISA.

### Radiological analysis

X-ray scanning was performed to evaluate the process of bone defect healing at four time points including the day of surgery, 7, 14, and 21 days after the surgery. All rats were anesthetized with isoflurane gas and then X-rays were taken (irradiation voltage: 48 kV, irradiation current: 100 mA, irradiation volume: 8 mAs, irradiation time: 0.06 s, and a distance between the X-ray tube and tibia: 59 cm). All radiographic outcomes were evaluated in randomized and double-blind conditions, and scored using the Lane-Sandhu scoring system [16] (Additional file 1: Table S1).

### Micro-computed Tomography analysis

To quantitatively assess bone formation within the defects, the specimens were scanned using a SkyScan 1176 high-resolution scanner (Brucker, Billerica, MA). The micro-CT instrument settings and bone morphometric parameter analysis protocol are described in Additional file 1: Section S1.

### Histopathological staining and analysis

Bone tissues were decalcified using 10% EDTA acid for 3 weeks after fixation in 10% buffered formalin for 48 h. EDTA solution was renewed every 3 days for the best decalcification efficacy. Bone tissues were then processed through an automatic tissue processor and paraffin-embedded. 4-µm-thick sections were cut from the paraffin-embedded tissues and subjected to hematoxylin and eosin (HE) staining.

 All sections were assessed by two experienced observers who were blinded to the background of this study and scored using the Lane-Sandhu histopathology scoring criteria [16] (Additional file 1: Table S2).

### Enzyme-linked immunosorbent assay

Rat tibia specimens were removed from − 80 °C, the injury area was clipped, the surrounding soft tissues were removed and rinsed with PBS, and then the bone tissues were grinded, ultrasonically crushed, and lysed in RIPA buffer (containing phosphatase inhibitor, protease inhibitor, PMSF) for 30 min on ice, and then centrifuged at a low temperature and high speed (4 °C, 12,000 rpm) for 15 min, and the supernatant of bone tissues were separated to test the levels of bone metabolism related parameters including bone-specific alkaline phosphatase (BALP), procollagen type I N-terminal propeptide (P1NP), bone morphogenetic protein-2 (BMP-2), transforming growth factor-β (TGF-β), serum calcium (Ca), serum phosphorus (P), and the enzyme activities of receptor-interacting serine/threonine-protein kinase 1 (RIPK1), receptor-interacting serine/threonine-protein kinase 3 (RIPK3) and mixed-lineage kinase domain-like protein (MLKL). Meanwhile, peripheral blood serum was utilized to detect the levels of inflammatory factors including tumor necrosis factor (TNF-α), interleukin-1β (IL-1β), interleukin-6 (IL-6). All of the above indicators were detected by ELISA kits (ml003415, ml038224, ml220061, ml690339, ml076827, ml323031, ml975369, ml685462, ml002859, ml102828, ml037361, Shanghai Enzyme Linked Biotechnology Co., Ltd.) following the corresponding manufacturer’s protocols.

### Gene expression profiling

The whole blood samples and the affected right tibia tissues collected from rats in different groups were used to detect the gene expression profiling which may represent the overall status of the body and the pathological characteristics of the affected tissues during bone defect development and progression by RNA-seq and microarray. DEGs among different groups were identified by the edge R package with *P* value < 0.05 and fold change >2/<0.5. Among them, the bone defect-related genes (DEGs of bone defect model group vs. normal control group) and Osteoking effective genes (DEGs of Osteoking high dose treatment group vs. bone defect model group) were identified and provided in Additional file 1: TableS3.

### Network construction and analysis

The differentially expressed genes in both the whole blood samples and the affected bone tissues obtained from RNA-seq and microarray detection were used for illustrating their functional interactions and evaluating their co-expression correlation. Briefly, the functional interactions among bone defect-related genes and Osteoking effective genes were collected from the public database STRING (version 12.0, http://cn.string-db.org/) with the combined score greater than 0.7 (high confidence), and bone defect-related genes and Osteoking effective genes with differential expression characteristics in the network were selected. After that, Pearson correlation analysis were performed on the fragments per kilobase of exon model per million mapped fragments (FPKM) for these genes in whole blood and bone tissue samples, and the Pearson correlation coefficients were set to be greater than 0.7 or less than − 0.7, as described in Additional file 1: Table S4. In addition, the network targets with both functional interactions and significant co-expression correlations were selected for pathway enrichment analysis based on Kyoto Encyclopedia of Genes and Genomes (KEGG, version 107.1, https://www.genome.jp/kegg/) and SoFDA (version 1.0, http://www.tcmip.cn/Syndrome/front/#/) [17].

### Immunohistochemical & immunofluorescence analyses

To detect the expression levels of phospho-RIPK1 (p-RIPK1), phospho-RIPK3 (p-RIPK3), phospho-MLKL (p-MLKL) and phospho-STAT1 (p-STAT1) in the callus of rats in bone defect rats and corresponding treatment groups, immunohistochemical staining and immunofluorescence analyses were carried out according to the routine protocols. Both immunohistochemistry and immunofluorescence quantification were performed using ImageJ software (version 1.51, https://imagej.nih.gov/ij/). The detailed protocols are provided in Additional file 1: Section S2 and the detailed information of antibodies is provided in Additional file 1: Table S5.

### Western blotting

To evaluate the regulatory effects of Osteoking on the expression levels of ZBP1, phospho-PKR (p-PKR) and PKR proteins in the callus of different groups, western blotting analysis was performed according to the protocol described in our previous studies [18–20]. β-actin was used as a loading control for bone tissue samples. Detailed information of antibodies is provided in Additional file 1: Table S5.

### Statistical analyses

All data analyses were performed using GraphPad Prism software (version 8.0.2, GraphPad Software, La Jolla, CA). All representative experiments in the current study were repeated at least three times. Data are presented in result and figures as mean ± SD. One-way ANOVA was used for comparisons among multiple groups, followed by Bonferroni’s or Dunnett’s post hoc tests. Results with *P* value less than 0.05 was deemed statistically significant.

## Results

### Osteoking promotes bone defect repair in tibia drill hole rats

**Fig. 1:**
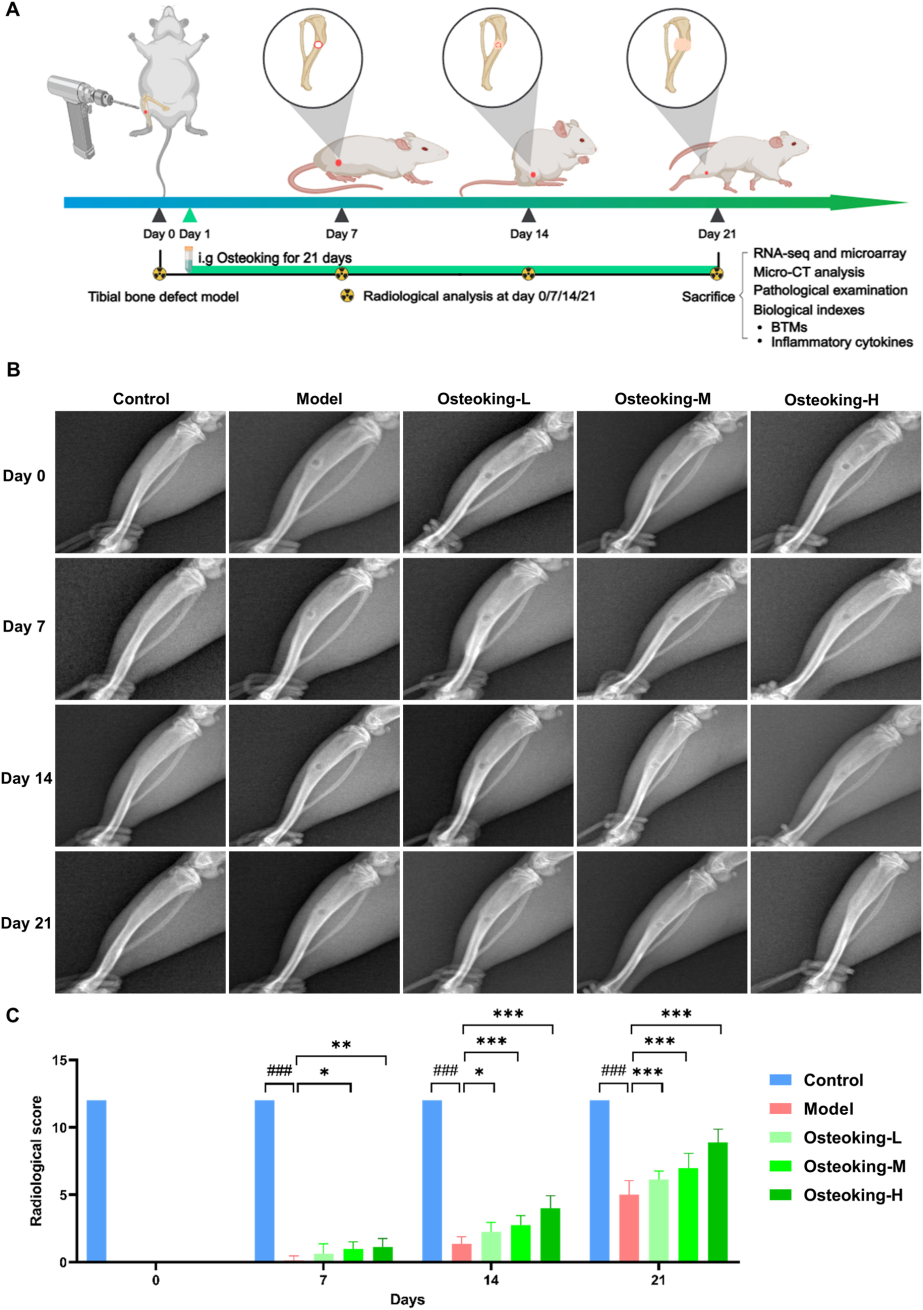
Evaluation of pharmacological effects of Osteoking in tibial bone defect repair of rats based on X-ray scanning. **A** Establishment of the tibial bone defect rat model, the dynamic repair process of bone defects, and schematic representation of the experimental design. **B** Representative X-ray scanning photographs of the affected bone tissues in different groups. **C** Radiological score of different groups were assessed based on the changes observed in X-ray scanning at different time points. Bars refer to mean ± standard deviation (SD). ^#^*P* < 0.05, ^##^*P* < 0.01 and ^###^*P* < 0.001 indicate the statistical significance compared to the normal control group, respectively; *P < 0.05, **P < 0.01 and ***P < 0.001 indicate the statistical significance compared to the model group, respectively

To determine the pharmacological effects of Osteoking against bone defect, we successfully established tibia drill hole rats (the achievement ratio of this model was 100%, Fig. 1A). The representative X-ray photographs of the right tibia scanned at all time points in each group showed the bone defects with uniform size, clear defect edges and no callus formation in all groups on the day of surgery. On the 7th day after the surgery, no change was observed in the bone defects of model group, while the callus of all Osteoking treatment groups showed different degrees of thickening. On the 14th day after the surgery, the callus of model rats was slightly thickened, but the size of the defects had not changed and the edges of the defects were still clearly visible. In contrast, the bone defects in all Osteoking treatment groups were significantly diminished, the edges of the defects were blurred, and the callus was significantly thickened, which was the most significant in the high-dose group. On the 21st day after the surgery, the bone defects in the model group were blurred, and the area of the defects filled with callus was about half of the initial defect area, while the bone defects in Osteoking treatment groups were significantly diminished, and the edges of the defects were partially present, especially in the high-dose group, where the callus was about to fill up the entire defect, and the edges of the defects had almost disappeared (Fig. 1B). Of note, the statistical results based on the Lane-Sandhu scores indicated that Osteoking treatment effectively improved the X-ray scores of bone defect rats, and the high dose Osteoking treatment group exerted the most significant pharmacological effects (*P* < 0.001, Fig. 1C).

### Osteoking accelerates the repair of cortical bone and promotes the growth of trabeculae in rats with bone defects

**Fig. 2:**
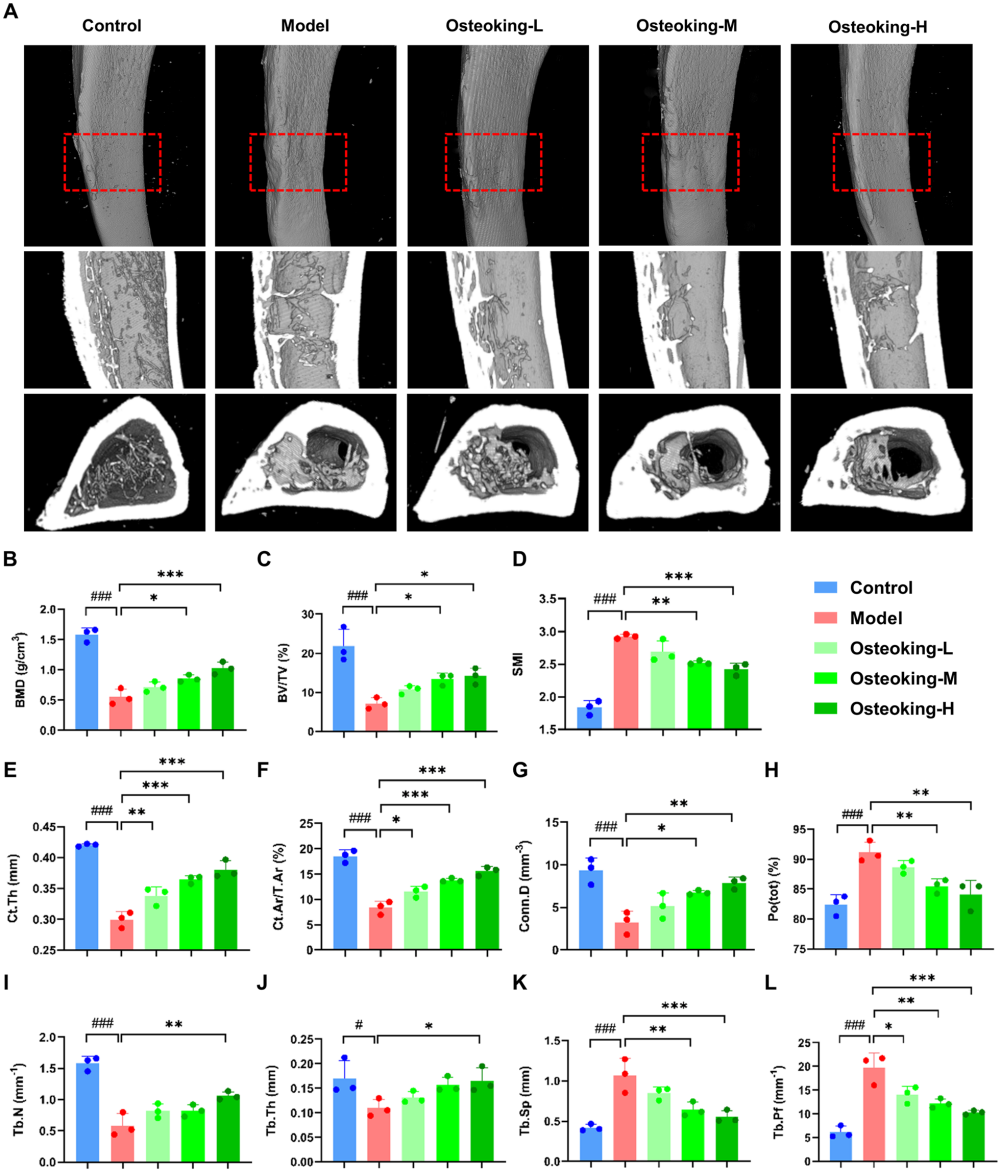
Evaluation on the bone formation and bone defect repair of rats in different groups based on Micro-CT analysis. **A** Representative 3D images of rat tibia with both exterior and interior general view at the 21st day post-surgery. **B**–**L** Results of bone morphometric parameters analysis (ANOVA, followed by Bonferron’s post hoc test; bars show mean ± SD; ^#^*P* < 0.05, ^##^*P* < 0.01 and ^###^*P* < 0.001 indicate the statistical significance compared to the normal control group, respectively; *P < 0.05, **P < 0.01 and ***P < 0.001 indicate the statistical significance compared to the model group, respectively). **B** Bone mineral density (BMD; g/cm^3^). **C** Bone volume/tissue volume ratio (BV/TV; %). **D** Structural model index (SMI). **E** Cortical bone thickness (Ct.Th; mm). **F** Cortical bone cross-sectional area ratio (Ct.Ar/T.Ar; %). **G** Connectivity density (Conn.D; mm^−3^). **H** Total porosity (Po(tot); %). **I** Trabecular number (Tb.N; mm^−1^). **J** Trabecular thickness (Tb.Th; mm). **K** Trabecular separation (Tb.Sp; mm). **L** Trabecular factor (Tb.Pf; mm^−1^)

The bone defect area was observed using micro-CT scanning after 21-day treatment with Osteoking to evaluate its pharmacological effects on bone repair (Fig. 2A). The exterior general view of the tibia displayed that the cortical bone was formed at the bone defect in all groups, but the surface of the tibia was still not smooth and flat, in which the obvious depression was shown in the model group, while the tibia surface of the Osteoking high-dose group had no obvious marks. In addition, the interior general views of the tibia revealed that the cortical bone at the bone defect in the model group had more gaps, poor connectivity and integrity, and sparse trabeculae in the marrow cavity, whereas the bone defect sites of the cortical and cancellous bone were demonstrated to be repaired to varying degrees by the treatment of Osteoking, especially the Osteoking high-dose group displayed the most intact cortical bone and the highest number of bone trabeculae in the marrow cavity.

Quantitatively, both BMD (model vs. control: 0.55 ± 0.13 vs. 1.58 ± 0.11, Fig. 2B) and BV/TV (model vs. control: 7.16 ± 1.47 vs. 21.84 ± 4.37, Fig. 2C) in the bone defect model group were markedly decreased, while both SMI (model vs. control: 2.92 ± 0.03 vs. 1.83 ± 0.11, Fig. 2D) and Tb.Pf (model vs. control: 19.68 ± 3.17 vs. 6.13 ± 1.26, Fig. 2L) were dramatically increased (all *P* < 0.001), which were all significantly reversed by the treatment of Osteoking with a dose-dependent manner. In addition, Ct.Th (model vs. control: 0.30 ± 0.01 vs. 0.42 ± 0.00, Fig. 2E), Ct.Ar/T.Ar (model vs. control: 8.36 ± 1.30 vs. 18.52 ± 1.21, Fig. 2F) and Conn.D (model vs. control: 3.24 ± 1.32 vs. 9.31 ± 1.52, Fig. 2G) in rats with the bone defect were all significantly lower than those in normal rats (all *P* < 0.001), while the values of Po(tot) in the model group were increased (model vs. control: 91.16 ± 1.72 vs. 82.38 ± 1.70, *P* < 0.001, Fig. 2H). Notably, Osteoking treatment effectively improved the thickness and connectivity of cortical bone and accelerated the repair of cortical bone by reversing the abnormal values of Ct.Th, Ct.Ar/T.Ar, Conn.D and Po(tot) (all *P* < 0.05). Moreover, the decreased Tb.N (model vs. control: 0.59 ± 0.19 vs. 1.58 ± 0.11, *P* < 0.001, Fig. 2I) and Tb.Th (model vs. control: 0.11 ± 0.02 vs. 0.17 ± 0.04, *P* < 0.05, Fig. 2J), and the increased Tb.Sp (model vs. control: 1.07 ± 0.21 vs. 0.42 ± 0.04, *P* < 0.001, Fig. 2K) in rats with bone defect were significantly recovered by the treatment of Osteoking (all *P* < 0.05).

### Osteoking promotes bone formation in rats with bone defects

**Fig. 3:**
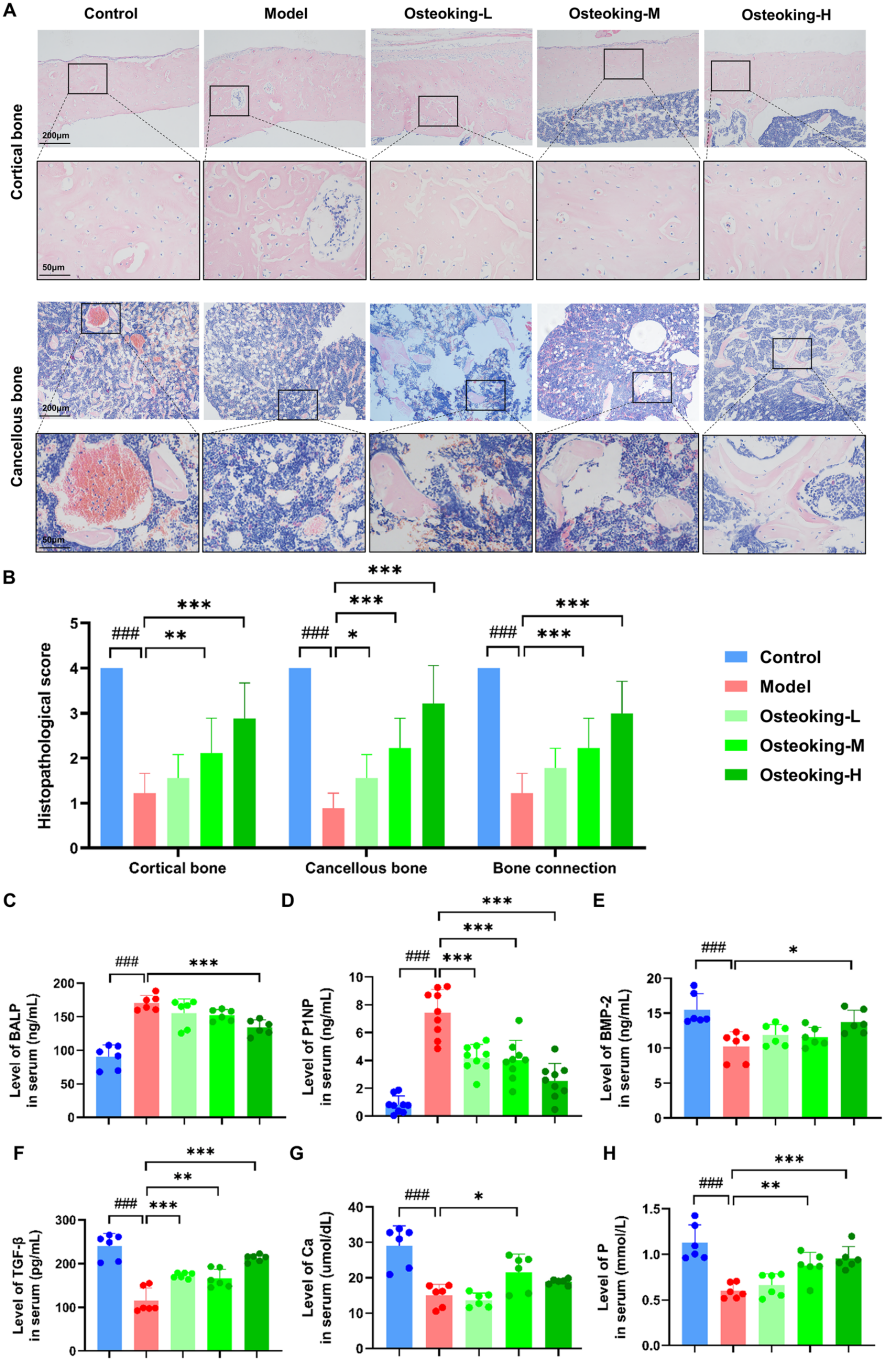
Pharmacological effects of Osteoking on the levels of histopathological and bone metabolism-related biochemical indicators. **A** Histopathological observations of different groups with hematoxylin and eosin (HE) staining after 21 days of Osteoking treatment. Original magnifications, ×100 and ×400; scale bars, 200 μm and 50 μm. **B** Histopathological score of different groups were assessed based on the changes observed in HE staining. **C** Bone-specific alkaline phosphatase (BALP, ng/mL). **D** Procollagen type 1 N-terminal propeptide (P1NP, ng/mL). **E** Bone morphogenetic protein-2 (BMP-2, ng/mL). **F** Transforming growth factor-β (TGF-β, pg/mL). **G** Serum calcium (Ca; µmol/dL). **H** Serum phosphorus (P; mmol/L). Bars show mean ± SD; ^#^*P* < 0.05, ^##^*P* < 0.01 and ^###^*P* < 0.001 indicate the statistical significance compared to the normal control group, respectively; *P < 0.05, **P < 0.01 and ***P < 0.001 indicate the statistical significance compared to the model group, respectively

To determine the roles of Osteoking in bone formation in vivo, we performed HE staining using the affected bone tissue sections collected from rats with bone defects after 21 days of drug treatment. In line with the micro-CT observation, the Osteoking treatment group displayed abundant area of osteoid and bone in the callus, and improved bone connectivity compared to the model group (Fig. 3A). Further histopathologic scores revealed that the bone defect model group had significantly lower scores in cortical bone, cancellous bone, and bone connection (all *P* < 0.001), and that Osteoking had a significant and dose-dependent therapeutic effect (Fig. 3B). Consistently, the abnormal levels of bone turnover markers (BTMs, BALP and P1NP), bone growth factors (BMP-2 and TGF-β) and bone metabolism-related biochemical indexes (Ca and P) in serum of rats with bone defects were also reversed by the treatment of Osteoking with a dose-dependent manner (Fig. 3C–H).

### Integration of transcriptomics profiling and network calculation reveals that Osteoking might attenuate ZBP1–STAT1–PKR–MLKL-mediated necroptosis in bone defect rats

**Fig. 4:**
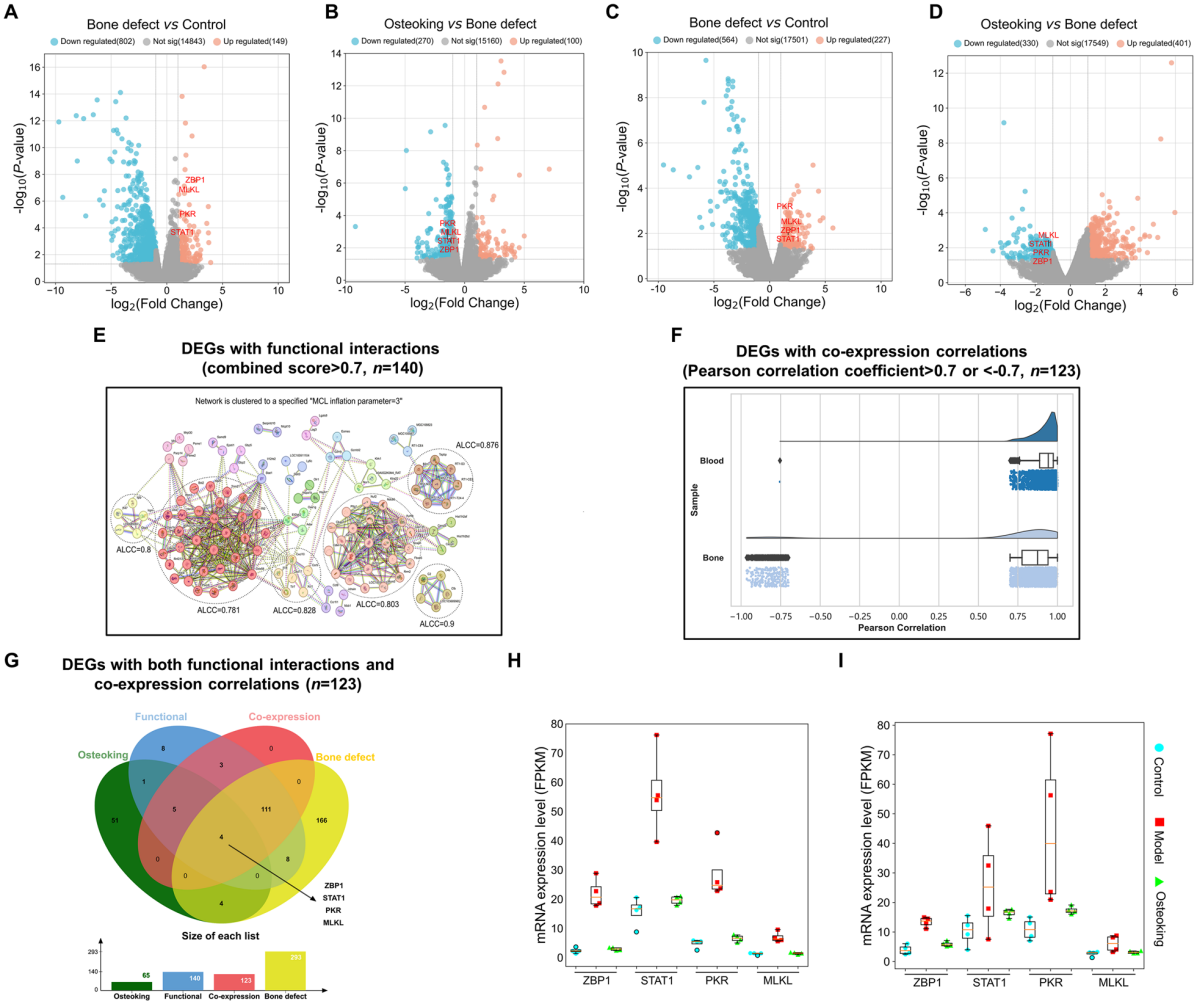
Transcriptomic profiling-based differential data analysis and biomolecular network-based investigation of candidate effective targets of Osteoking against bone defect. **A** The volcano plot of the DEGs between the bone defect model group and the normal control group in whole blood tissues using microarray analysis (*n* = 4 per group). **B** The volcano plot of the DEGs between the Osteoking treatment group and the bone defect model group in whole blood tissues using microarray analysis (*n* = 4 per group). **C** The volcano plot of the DEGs between the bone defect model group and the normal control group in bone tissues using RNA sequencing (RNA-Seq) analysis (*n* = 4 per group). **D** The volcano plot of the DEGs between the Osteoking treatment group and the bone defect model group in bone tissues using RNA-Seq analysis (*n* = 4 per group). **E** Functional interaction network analysis of differentially expressed genes in whole blood and bone tissues. The Markov cluster algorithm (MCL). Average local clustering coefficient (ALCC). **F** Co-expression correlation calculation of differentially expressed genes in whole blood and bone tissues. **G** The Venn diagram of the DEGs with both functional interactions and co-expression correlations. **H** Expression levels of core differentially expressed genes (ZBP1, STAT1, PKR, MLKL) in whole blood samples from normal control group, bone defect model group and Osteoking treatment group. **I** Expression levels of core differentially expressed genes (ZBP1, STAT1, PKR, MLKL) in bone samples from normal control group, bone defect model group and Osteoking treatment group

To determine the bone defect-related genes and the effective targets of Osteoking against this disease, the transcriptomic profiling of the whole blood and the affected bone tissues collected from rats in different groups were identified. There were a total of 293 bone defect-related genes (DEGs in comparison between bone defect model group vs. normal control group, including 31 upregulated and 262 downregulated genes) and 65 Osteoking effective genes (DEGs in comparison between Osteoking high dose treatment group vs. bone defect model group, including 16 upregulated and 49 downregulated genes) as shown in Additional file 1: Table S2. In addition, the “disease gene-drug effective target” interaction network was constructed based on links between 293 bone defect-related genes and 65 Osteoking effective genes. After the calculation of the coexpression correlation among the above network node genes, 123 major network targets including 115 bone defect-related genes and 9 Osteoking effective genes with significant coexpression correlations were screened for the following functional analysis (Fig. 4E–G).

**Fig. 5:**
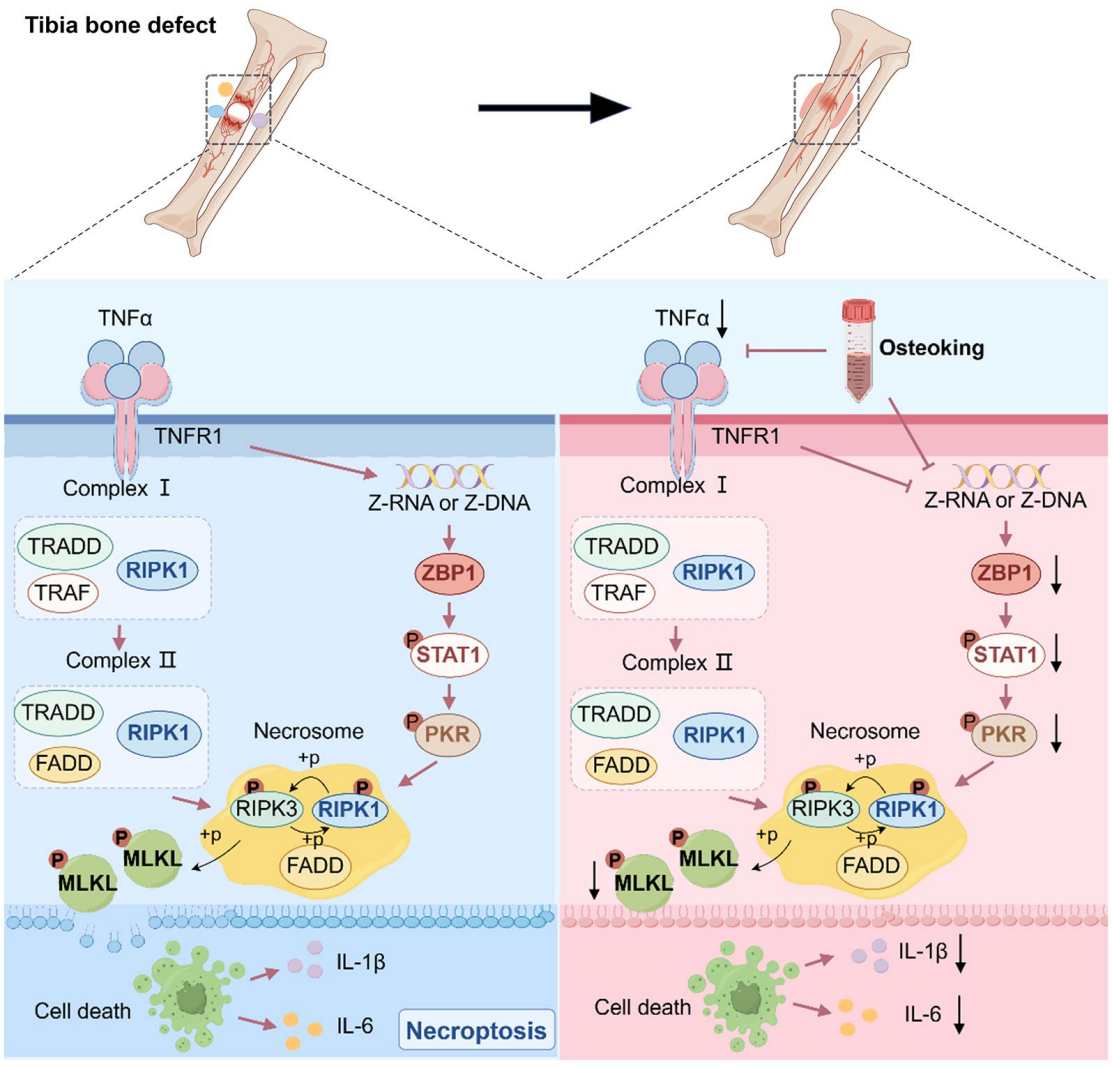
The schematic diagram of the underlying mechanism of Osteoking promoting bone defect repair and attenuating inflammatory response by inhibiting necroptosis induced by ZBP1–STAT1–PKR–MLKL signaling axis. “→”: Activation. “┴”: Inhibition

Functionally, the above major network targets were significantly involved into various cellular function and immune system regulation-related pathways, such as cell cycle, cellular senescence, necroptosis, NOD-like receptor signaling, Toll-like receptor signaling, antigen processing and presentation, cytosolic DNA-sensing, RIG-I-like receptor signaling, and etc. according to the pathway enrichment analysis. Especially, four Osteoking effective genes (ZBP1, STAT1, PKR and MLKL) with both functional interactions and co-expression correlations, and their interaction partners (RIPK1 and RIPK3), which are key upstream molecules of necroptosis, formed a signal axis which might play an essential role in the progression and intervention of bone defect. Notably, the mRNA expression levels of ZBP1, STAT1, PKR and MLKL were all upregulated in bone defect rats compared to normal control rats, while downregulated by Osteoking treatment based on the transcriptomic profiling (Fig. 4H and I). Necroptosis is a regulated form of inflammatory cell death, which is induced by a variety of extracellular or intracellular stimuli, with TNF-α and ZBP1-induced necroptosis being the most characterized, respectively. After the occurrence of bone defect, local tissue damage and the release of inflammatory mediators activate the tumor necrosis factor receptor (TNFR) and its functional correlates, which subsequently induces the assembly of two typical necrosome complexes consisting of RIPK1, and finally interacts with RIPK3 and MLKL to form necrosome complex that mediate necroptosis. Meanwhile, mechanistic studies have shown that TNF-α induces severe DNA damage [21–23], leading to leakage of genomic DNA into cytoplasm, consequently activating the innate immunoreceptor ZBP1, which subsequently forms homotypic interactions with the RHIM structural domain-containing proteins RIPK1 and RIPK3, and in turn promotes the phosphorylation of STAT1. Phosphorylated STAT1 undergoes dimerization, which triggers the transcription and activation of PKR, further contributing to the formation of a high-density punctate complex between ZBP1, RIPK1, RIPK3, and MLKL, which mediates cell death [24–29]. Of note, recent studies have shown that the activation of PKR not only increases the production of inflammatory factors such as IL-6 and TNF-α, but also increases the degradation of bone matrix, leading to bone destruction [30–32]. After necroptosis occurs, cell membranes rupture and organelles are lysed and effluxed, resulting in the release of inflammatory factors, leading to a localized inflammatory infiltrate in the bone defects and inhibiting the proliferation of osteoblasts, which hampers bone formation and the repair of bone defects [33–35]. On this basis, we hypothesized that Osteoking might promote bone formation and bone defect repair via regulating ZBP1–STAT1–PKR–RIPK1–RIPK3–MLKL signaling-mediated necroptosis (Fig. 5).

### Experimental validation demonstrates that Osteoking suppresses ZBP1–STAT1–PKR–MLKL-mediated necroptosis activated in bone defect rats

**Fig. 6:**
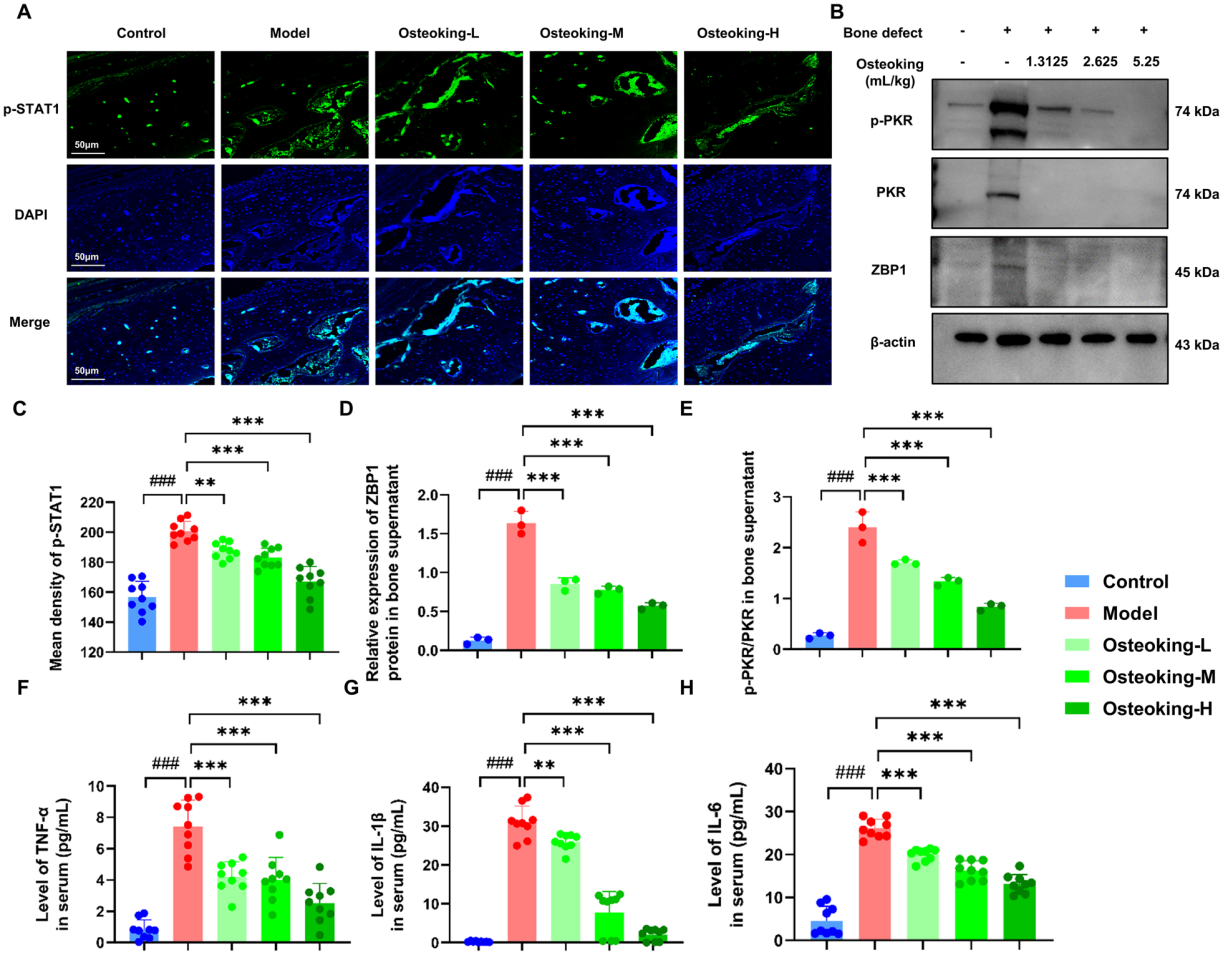
Regulatory effects of Osteoking on the expression of the corresponding proteins in ZBP1-STAT1-PKR-MLKL signal axis in bone tissues of bone defect rats. **A** The expression levels of p-STAT1 protein were measured by immunofluorescence staining and confocal microscopy in bone tissue sections (p-STAT1 green, DAPI blue; Original magnification, ×400; scale bar, 50 μm). **B** The expression levels of ZBP1 and p-PKR/PKR proteins in bone tissues of bone defect rats using Western botting analysis (*n* = 3 per group). **C** Mean density of immunofluorescence staining of p-STAT1 protein expression in the bone tissues of different groups. **D** The expression levels of ZBP1 in bone tissue supernatants were measured by Western blotting. **E** The expression levels of p-PKR in bone tissue supernatants were measured by Western blotting. **F**–**H** The expression levels of inflammatory factors in serum were detected by ELISA. **F** Tumor necrosis factor (TNF-α; pg/mL). **G** Interleukin-1β (IL-1β; pg/mL). **H** Interleukin-6 (IL-6; pg/mL). Bars show mean ± SD; ^#^*P* < 0.05, ^##^*P* < 0.01 and ^###^*P* < 0.001 indicate the significance compared to the normal control group, respectively; *P < 0.05, **P < 0.01 and ***P < 0.001 indicate the significance compared to the model group, respectively

To verify the regulatory effects of Osteoking on the ZBP1–STAT1–PKR–RIPK1/RIPK3–MLKL signaling axis, the expression levels of upstream and downstream proteins of necroptosis were detected by immunofluorescence, western blotting and ELISA, respectively. The results showed that ZBP1, STAT1 and PKR were highly expressed in the bone defect model group compared with the normal control group (Fig. 6A–E), and the expression of the hallmark inflammatory cytokines for the end of necroptosis TNF-α, IL-1β and IL-6 was also significantly increased (Fig. 6F–H), which were all effectively reversed by the treatment of Osteoking.

**Fig. 7:**
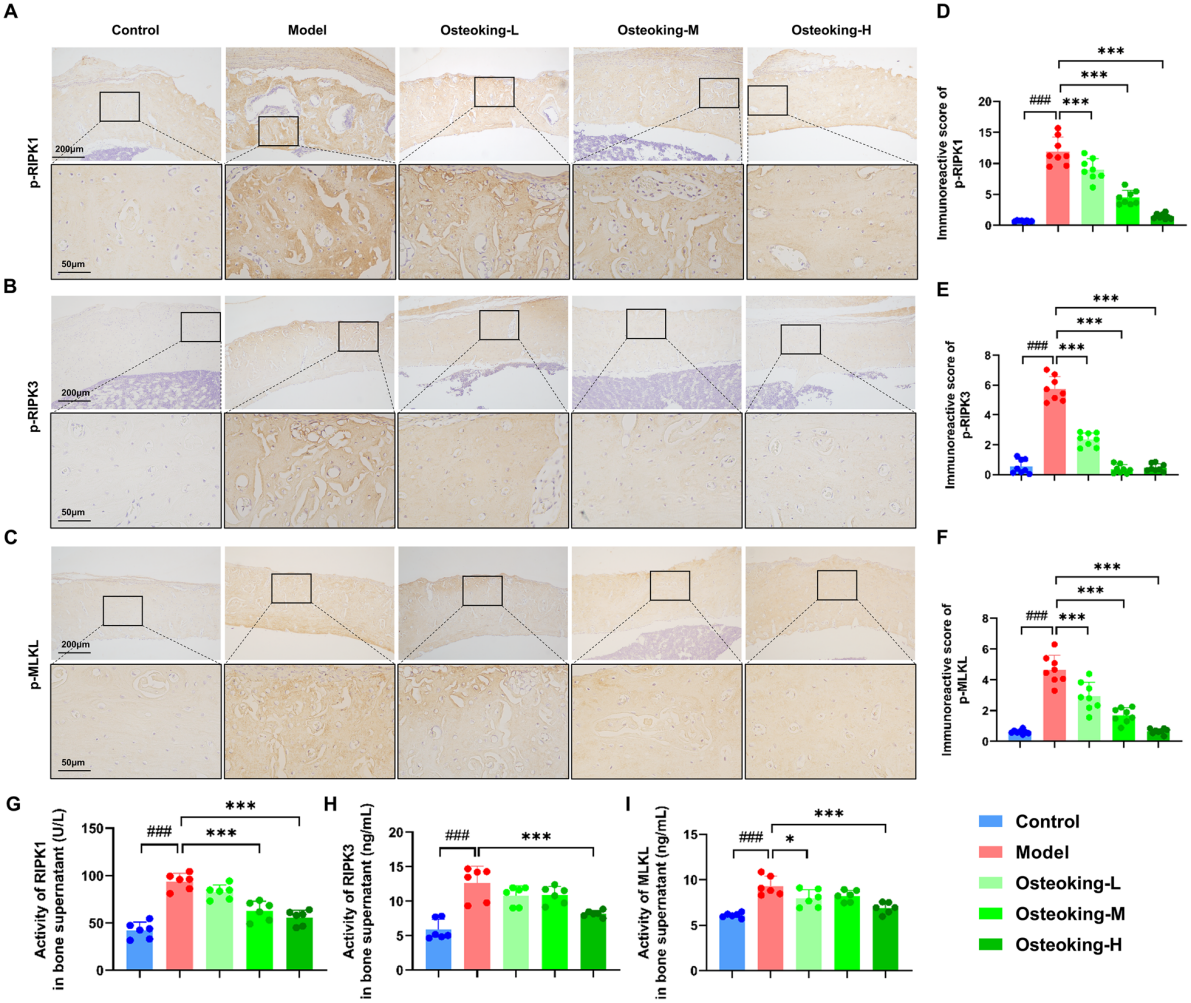
Regulatory effects of Osteoking on the expression of key proteins for necroptosis in bone tissues of bone defect rats. **A**–**C** The expression levels of p-RIPK1, p-RIPK3, p-MLKL proteins in bone tissue of rats in different groups detected by immunohistochemical assay. Original magnifications, ×100 and ×400; scale bars, 200 μm and 50 μm. **D**–**F** Immunoreactive scores of p-RIPK1, p-RIPK3, p-MLKL proteins expression levels in bone tissues of rats in different groups. **G**–**I** The activity of key proteins for necroptosis in bone tissue supernatants were detected by ELISA. **G** Receptor-interacting serine/threonine-protein kinase 1 (RIPK1; U/L). **H** Receptor-interacting serine/threonine-protein kinase 3 (RIPK3; ng/mL). **I** Mixed-lineage kinase domain-like protein (MLKL; ng/mL). Bars show mean ± SD; ^#^*P* < 0.05, ^##^*P* < 0.01 and ^###^*P* < 0.001 indicate the significance compared to the normal control group, respectively; *P < 0.05, **P < 0.01 and ***P < 0.001 indicate the significance compared to the model group, respectively

After that, the expression levels of necroptosis markers (p-RIPK1, p-RIPK3 and p-MLKL) were detected by immunohistochemistry and ELISA, respectively. The immunohistochemical results showed that the protein expression levels of p-RIPK1, p-RIPK3 and p-MLKL were markedly increased in the bone defect rats, which were effectively decreased by the treatment with Osteoking (all *P* < 0.001, Fig. 7A–F). Moreover, data obtained from ELISA demonstrated the suppressive effects of Osteoking on the activities of RIPK1, RIPK3 and MLKL in the bone supernatants which were abnormally activated in bone defect rats (Fig. 7G–I).

## Discussion

Research and development of the optimal treatment for fractures and bone defects may be a great clinical challenge for orthopedics and related specialties. In the current study, we established the bone defect rat model to evaluate the pharmacological effects of Chinese Patent Drug Osteoking, and found that this prescription effectively promoted bone defect repair in rats by accelerating the repair of cortical bone and the growth of trabeculae. Histopathologically, the bone defect rats displayed lower histopathologic scores in cortical bone, cancellous bone and bone connection than normal controls, and Osteoking exerted a significant and dose-dependent therapeutic effect. Consistently, the abnormal levels of bone turnover markers, bone growth factors and bone metabolism-related biochemical indexes in serum of bone defect rats were also reversed by the treatment of Osteoking with a dose-dependent manner. Following the transcriptome-based network investigation, we hypothesized that osteoking might attenuate the levels of ZBP1–STAT1–PKR–MLKL-mediated necroptosis involved into bone defect. Experimentally, the expression levels of ZBP1, STAT1, PKR and the hallmark inflammatory cytokines for the end of necroptosis were distinctly elevated in bone defect rats, but were all effectively reversed by the treatment of Osteoking. Moreover, the activities of RIPK1, RIPK3 and MLKL in bone tissue supernatants were also effectively suppressed by the treatment of Osteoking. To the best of our knowledge, this is the first study to identify necroptosis as one of the potential effective targets of Osteoking against bone defects.

Osteoking, originated from the Yi ethnic group within the Yunnan Province of China [36], has been revealed to play a role in improving blood circulation and providing nutritional support to the liver and the kidneys [9]. Accumulating studies have indicated that this prescription may have a potential in impeding cartilage degeneration, increasing BMD and promoting calcium deposition [11, 12], which were in accord to our radiological and histopathological observations. In addition, we found that the reversal of abnormal expression of bone formation markers such as BALP, P1NP, BMP2, and TGF-β may contribute to the beneficial impact of Osteoking on the maturation of osteoblasts, consistent with the previous reports [12, 37]. Our data revealed that the pharmacological effects of Osteoking were in accord to the TCM theory of “kidney governs bone”.

There are three partially overlapping phases during bone healing, including the inflammatory phase, the endochondral bone formation phase, and the coupled remodeling phase [38]. Growing evidence show that the eventual bone healing may be highly dependent on the initial inflammatory phase, which may be influenced by both the local and systemic responses to the injurious stimulus [39–41]. Necroptosis is one of various ways to the demise of cells distinguished by an unprogrammed and passive mechanism, commonly initiated by external factors such as damage, infection, ischemia, and similar stressors [42, 43]. It also involves a sequence of events, specifically the perturbation of the cellular membrane, extracellular release of intracellular components, and subsequent activation of damage-associated molecular patterns (DAMPs), and inflammatory cytokines, to trigger inflammation [44]. Necroptosis is characterized by the initiation of the RIPK1–RIPK3–MLKL necrosome complex cascade, leading to the disruption of the plasma membrane integrity and subsequent liberation of immunostimulatory intracellular components [45]. Interestingly, several recent studies have revealed that RIPK1–RIPK3–MLKL signaling-mediated necroptosis may be involved into fractures, specifically impacting the viability of osteoblasts, osteoclasts, and chondrocytes, therefore leading to a prolongation in the healing process of bones [33, 46, 47].

Accordingly, our transcriptome-based network investigation hypothesized that the pharmacological effects of Osteoking in promoting bone formation and bone defect repair might be associated with its inhibitory roles in necroptosis occurred in the affected bone tissues by regulating the potential effective targets. Among them, ZBP1 and PKR are two distinct innate immunoreceptors and crucial components in the necroptosis that exhibit the ability to detect diverse forms of DNA or RNA molecules, thereby eliciting distinct immunological reactions and initiating the immune cell’s inflammatory response [28, 48]. Similar to RIPK1 and RIPK3, ZBP1 is a protein that contains a RHIM (RIP homotypic interaction motif) domain [49]. Upon binding and sensing Z-nucleic acids, ZBP1 initiates the activation of RIPK3, which in turn phosphorylates MLKL, resulting in the disruption of membrane integrity and induction of necroptosis [50]. Notably, ZBP1 serves as an essential part in the stabilization of Z-form mitochondrial DNA and the initiation of a cytosolic complex that consists of RIPK1 and RIPK3, which in turn supports the sustained phosphorylation of STAT1 [25]. PKR acts as a vital sensor of the innate immune system within the cytoplasmic environment which is widely expressed in various cell lineages and demonstrates activation in response to stress stimuli, double-stranded RNA, viral pathogens, and pro-inflammatory cytokines [51]. It operates as a pivotal regulator of transcription, translation, cellular proliferation, cellular differentiation, metabolism, and apoptosis. The role of PKR is of significant importance in the process of interferon-induced necroptosis mediated by RIPK1 and RIPK3, as indicated by previous studies [28]. The activation of PKR by interferons occurs through transcriptional mechanisms, specifically in a JAK1/STAT1 dependent manner. The activation of PKR subsequently triggers the assembly of the necrosome complex, which is composed of RIPK1 and RIPK3. After that, they initiate the activation of the pro-necroptotic protein MLKL through the process of phosphorylation, consequently inducing necroptotic plasma membrane permeabilization [27, 52]. In line with the above regulatory mode, our experimental data in vivo verified that the abnormal upregulation of ZBP1, STAT1 and PKR proteins, and the elevated levels of the hallmark inflammatory cytokines for the end of necroptosis TNF-α, IL-1β and IL-6 in the affected bone tissues of bone defect rats were all significantly reversed by the treatment of Osteoking. Consistently, the abnormally activated necroptosis marker proteins RIPK1, RIPK3 and MLKL in the bone defect model group were also effectively inhibited by this drug treatment.

In conclusion, our data suggest that the activation of necroptosis may exist in the affected bone tissues of bone defect rats, which may be closely associated with the pathogenesis of this disease. The pharmacological mechanisms of Osteoking in promoting bone formation and bone defect repair may be associated with the regulation of multiple targets in necroptosis, implying that necroptosis may be a new potential target for the treatment of fractures and bone defects. However, the pharmacological mechanisms of Osteoking against pain caused by fractures are also need to be investigated in our further research.

### Supplementary Information

**Additional file 1: Section S1.** Micro-computed tomography (micro-CT) analysis. **Section S2.** Immunohistochemical& Immunofluorescence analysis. **Table S1.** Lane-Sandhu X-ray scoring system. **Table S2.** Lane-Sandhu histopathology scoring system. **Table S3.** The differentially expressed genes (DEGs) identified by microarray. **Table S4.** The differentially expressed genes (DEGs) with both functional interactions and significant co-expression correlations. **Table S5.** The antibodies used for immunohistochemistry staining, Immunofluorescence analysis and western blotting.

## Data Availability

The data supporting the results are included in this published article and in Additional materials.
