# Supplementary material for: Osteoking promotes bone formation and bone defect repair through ZBP1–STAT1–PKR–MLKL-mediated necroptosis
Source: Chin Med. 2024 Jan 18;19:13. doi: 10.1186/s13020-024-00883-4 (PMC10797925; doi:10.1186/s13020-024-00883-4)
Supplement: Supplementary file 1 — Additional file 1: Section S1. Micro-computed tomography (micro-CT) analysis. Section S2. Immunohistochemical& Immunofluorescence analysis. Table S1. Lane-Sandhu X-ray scoring system. Table S2. Lane-Sandhu histopathology scoring system. Table S3. The differentially expressed genes (DEGs) identified by microarray. Table S4. The differentially expressed genes (DEGs) with both functional interactions and significant co-expression correlations. Table S5. The antibodies used for immunohistochemistry staining, Immunofluorescence analysis and western blotting. [file 13020_2024_883_MOESM1_ESM.docx]

**Additional Materials for “Osteoking Promotes Bone Formation and Bone Defect Repair through Regulating ZBP1-STAT1-PKR-MLKL-mediated Necroptosis”**

**List of Additional Materials**

Section S1 Micro-computed tomography (micro-CT) analysis

Section S2 Immunohistochemical & Immunofluorescence analysis

Table S1 Lane-Sandhu X-ray scoring system

Table S2 Lane-Sandhu histopathology scoring system

Table S3 The differentially expressed genes (DEGs) identified by microarray

Table S4 The differentially expressed genes (DEGs) with both functional interactions and significant co-expression correlations.

Table S5 The antibodies used for immunohistochemistry staining, Immunofluorescence analysis and western blotting.

**Section S1 Micro-computed tomography (micro-CT) analysis**

To quantitatively assess bone formation within the defects, the specimens were scanned using a SkyScan 1176 high-resolution scanner (Brucker, Billerica, MA) with 70 kV voltage and 114 μA current at a 15.6 μm/pixel resolution. Whole-scanned region was included as the volume of interest (VOI) from the bone defect model to generate a general 3D view of the tibia injury site by CTvox (version 2.4.0, SkyScan). In order to analyze only the callus bone parameters, a total of 4 mm (240 transverse anatomic slides) radius including the entire injury site was defined as the region of interest (ROI), and CTAn (version 1.12.10.0, SkyScan) was used for quantitative analysis to calculate the following bone morphometric parameters: bone mineral density (BMD), relative bone volume (BV/TV), trabecular number (Tb.N), trabecular thickness (Tb.Th), trabecular separation (Tb.Sp), trabecular pattern factor (Tb.Pf), structural model index (SMI), total cortical bone area (T.Ar), cortical bone area (Ct.Ar), cortical bone thickness (Ct.Th), connectivity density (Conn.D).

**Section S2 Immunohistochemical & Immunofluorescence analysis**

The immunohistochemical staining was conducted using an UltraSensitive SP

(mouse/rabbit) immunohistochemistry kit (KIT-9707, MX Biotechnologies, Fuzhou,

China), which contained endogenous peroxidase blocking solution, serum, secondary

antibody, streptavidin-peroxidase, and diaminobenzidine (DAB) substrate-chromogen

(DAB-0031, MX Biotechnologies, Fuzhou, China), and the primary antibodies to p-RIPK1, p-RIPK3 and p-MLKL were used. Immunofluorescence was performed using p-STAT1 primary antibody. Sections were then labeled with FITC-conjugated secondary antibody for 1 h at room temperature, avoiding light, and counterstained with 4′,6-diamidino-2-phenylindole (DAPI, G1012, Servicebio, Wuhan, China) staining solution for 4 min at room temperature. Subsequently, the sections were sealed with an anti-fluorescence quencher. Fluorescence images were taken with a Newport RS 2000 confocal microscope. Both immunohistochemistry and immunofluorescence were quantified by calculating the product of the area and the average intensity of at least three high-power fields using ImageJ software.

**Table S1 Lane-Sandhu X-ray scoring system**

| **Bone formation** | **Bone connection** | **Bone shaping** | **Score** |
| --- | --- | --- | --- |
| Boneless formation | Clear fracture line | No bone shaping | 0 |
| Bone formation accounts for 25% of the defect | Clear fracture line | No bone shaping | 1 |
| Bone formation accounts for 50% of the defect | Fracture line partially present | Bone marrow cavity formation | 2 |
| Bone formation accounts for 75% of the defect | Fracture line partially present | Bone marrow cavity formation | 3 |
| Bone formation filled with defects | Fracture line disappears | Cortical bone shaping | 4 |

**Table S2 Lane-Sandhu histopathology scoring system**

| **Connection** | **Cancellous bone** | **Cortical bone** | **Score** | |
| --- | --- | --- | --- | --- |
| Connectionless | No osteoclastic activity | No cortical bone growth | 0 |  |
| Fiber connection | Early aggregation of new bone | Early manifestations of cortical bone growth | 1 |  |
| Osseous and osteoid connection | Aggregation of active new bone | Cortical bone is forming | 2 |  |
| Osseous connection | The cancellous bone is being remodeled | Cortical bone is mostly modified | 3 |  |
| Complete regeneration of the connecting backbone | The cancellous bone is fully formed | Complete bone regeneration in cortical bone | 4 |  |

**Table S3 The differentially expressed genes (DEGs) identified by microarray analysis**

| **Sample type** | **Group** | **Gene Symbol** | **Log_2_fold change** | **P-value** | **Regulation** |
| --- | --- | --- | --- | --- | --- |
| Blood | Bone defect *vs.* Control | *Tmcc2* | 3.350587808 | 9.27E-17 | UP |
| Blood | Bone defect *vs.* Control | *Oas1g* | -4.165640377 | 7.71E-15 | DOWN |
| Blood | Bone defect *vs.* Control | *Loc103690020* | 1.364418859 | 1.50E-14 | UP |
| Blood | Bone defect *vs.* Control | *Aabr07006278.1* | -6.260313445 | 2.78E-14 | DOWN |
| Blood | Bone defect *vs.* Control | *Oas1a* | -4.589692017 | 3.74E-14 | DOWN |
| Blood | Bone defect *vs.* Control | *Isg15* | -6.607367203 | 3.54E-13 | DOWN |
| Blood | Bone defect *vs.* Control | *Siglec1* | -8.135973809 | 4.18E-13 | DOWN |
| Blood | Bone defect *vs.* Control | *Spats2l* | -7.473699863 | 6.95E-13 | DOWN |
| Blood | Bone defect *vs.* Control | *Oas2* | -3.665544771 | 6.25E-13 | DOWN |
| Blood | Bone defect *vs.* Control | *Apol9a* | -9.701062551 | 1.22E-12 | DOWN |
| Blood | Bone defect *vs.* Control | *Aabr07059308.1* | 1.666116691 | 1.48E-12 | UP |
| Blood | Bone defect *vs.* Control | *Lgals3bp* | -4.50711508 | 4.89E-12 | DOWN |
| Blood | Bone defect *vs.* Control | *Irf7* | -4.783869891 | 5.31E-12 | DOWN |
| Blood | Bone defect *vs.* Control | *Mx2* | -4.390692828 | 9.13E-12 | DOWN |
| Blood | Bone defect *vs.* Control | *Loc100911804* | 2.260048192 | 1.38E-11 | UP |
| Blood | Bone defect *vs.* Control | *Usp18* | -4.030307863 | 2.23E-11 | DOWN |
| Blood | Bone defect *vs.* Control | *Oasl* | -3.411029909 | 4.19E-11 | DOWN |
| Blood | Bone defect *vs.* Control | *Dhx58* | -3.188979096 | 6.17E-11 | DOWN |
| Blood | Bone defect *vs.* Control | *Loc497796* | -2.529509727 | 6.90E-11 | DOWN |
| Blood | Bone defect *vs.* Control | *Xaf1* | -3.506734224 | 1.19E-10 | DOWN |
| Blood | Bone defect *vs.* Control | *Ifi27* | -3.232064012 | 1.73E-10 | DOWN |
| Blood | Bone defect *vs.* Control | *Slamf9* | -4.672561367 | 2.16E-10 | DOWN |
| Blood | Bone defect *vs.* Control | *Top2a* | -2.740960111 | 2.84E-10 | DOWN |
| Blood | Bone defect *vs.* Control | *Tspan8* | 1.714210828 | 3.71E-10 | UP |
| Blood | Bone defect *vs.* Control | *F10* | -5.110075932 | 7.23E-10 | DOWN |
| Blood | Bone defect *vs.* Control | *Ifit2* | -2.931901434 | 9.53E-10 | DOWN |
| Blood | Bone defect *vs.* Control | *Aa926063* | -8.044176296 | 1.02E-09 | DOWN |
| Blood | Bone defect *vs.* Control | *Mx1* | -2.832791571 | 1.14E-09 | DOWN |
| Blood | Bone defect *vs.* Control | *Slc27a3* | -4.925503201 | 1.12E-09 | DOWN |
| Blood | Bone defect *vs.* Control | *Rtp4* | -3.885288631 | 1.19E-09 | DOWN |
| Blood | Bone defect *vs.* Control | *Herc6* | -3.342914539 | 1.31E-09 | DOWN |
| Blood | Bone defect *vs.* Control | *Ifi44* | -2.918570334 | 1.56E-09 | DOWN |
| Blood | Bone defect *vs.* Control | *C2* | -3.448341581 | 1.64E-09 | DOWN |
| Blood | Bone defect *vs.* Control | *Cdkl1* | 1.627404754 | 4.47E-09 | UP |
| Blood | Bone defect *vs.* Control | *Ifit3* | -2.582654881 | 6.74E-09 | DOWN |
| Blood | Bone defect *vs.* Control | *Oas3* | -2.310099367 | 8.10E-09 | DOWN |
| Blood | Bone defect *vs.* Control | *Parp12* | -2.319946308 | 1.13E-08 | DOWN |
| Blood | Bone defect *vs.* Control | *Ly6c* | -2.406941034 | 1.22E-08 | DOWN |
| Blood | Bone defect *vs.* Control | *Fbn1* | -3.438258013 | 1.34E-08 | DOWN |
| Blood | Bone defect *vs.* Control | *Cxcl17* | -3.647767958 | 1.34E-08 | DOWN |
| Blood | Bone defect *vs.* Control | *Tor3a* | -2.610528545 | 1.46E-08 | DOWN |
| Blood | Bone defect *vs.* Control | *Mgc105567* | -2.597591068 | 1.86E-08 | DOWN |
| Blood | Bone defect *vs.* Control | *Ifi27l2b* | -2.237347243 | 2.30E-08 | DOWN |
| Blood | Bone defect *vs.* Control | *Bst2* | -2.637651078 | 2.29E-08 | DOWN |
| Blood | Bone defect *vs.* Control | *Zbp1* | 2.556265001 | 2.92E-08 | UP |
| Blood | Bone defect *vs.* Control | *Ube2l6* | -2.972775044 | 2.88E-08 | DOWN |
| Blood | Bone defect *vs.* Control | *Cdkn1a* | -2.508151223 | 3.32E-08 | DOWN |
| Blood | Bone defect *vs.* Control | *Aabr07025140.1* | -2.550679285 | 3.99E-08 | DOWN |
| Blood | Bone defect *vs.* Control | *Lgals9* | -3.056337316 | 5.11E-08 | DOWN |
| Blood | Bone defect *vs.* Control | *Ifih1* | -1.996524406 | 5.55E-08 | DOWN |
| Blood | Bone defect *vs.* Control | *Xpo7* | 1.558165422 | 7.54E-08 | UP |
| Blood | Bone defect *vs.* Control | *Aabr07044322.1* | -2.666337495 | 8.19E-08 | DOWN |
| Blood | Bone defect *vs.* Control | *C4b* | -3.279877129 | 9.96E-08 | DOWN |
| Blood | Bone defect *vs.* Control | *Slfn4* | -2.249792855 | 1.12E-07 | DOWN |
| Blood | Bone defect *vs.* Control | *Samd9* | -2.811174708 | 1.17E-07 | DOWN |
| Blood | Bone defect *vs.* Control | *Aabr07044364.1* | -3.301137768 | 1.26E-07 | DOWN |
| Blood | Bone defect *vs.* Control | *Aabr07051562.1* | -3.881668532 | 1.35E-07 | DOWN |
| Blood | Bone defect *vs.* Control | *Mtmr7* | -1.823854815 | 1.39E-07 | DOWN |
| Blood | Bone defect *vs.* Control | *Mlkl* | 2.005316789 | 1.54E-07 | UP |
| Blood | Bone defect *vs.* Control | *Rnf114* | -2.502939125 | 1.67E-07 | DOWN |
| Blood | Bone defect *vs.* Control | *Sectm1b* | -5.608554846 | 1.72E-07 | DOWN |
| Blood | Bone defect *vs.* Control | *Rt1-T24-3* | -3.202817113 | 1.91E-07 | DOWN |
| Blood | Bone defect *vs.* Control | *Znfx1* | -2.344152702 | 1.98E-07 | DOWN |
| Blood | Bone defect *vs.* Control | *Oasl2* | -2.402912414 | 2.27E-07 | DOWN |
| Blood | Bone defect *vs.* Control | *Fam111a* | 1.571301113 | 2.67E-07 | UP |
| Blood | Bone defect *vs.* Control | *Gzmb* | -2.364217042 | 3.29E-07 | DOWN |
| Blood | Bone defect *vs.* Control | *Rnf213* | -2.070475381 | 3.69E-07 | DOWN |
| Blood | Bone defect *vs.* Control | *Aabr07006275.1* | -2.932397261 | 3.69E-07 | DOWN |
| Blood | Bone defect *vs.* Control | *Uba7* | -1.914401063 | 5.00E-07 | DOWN |
| Blood | Bone defect *vs.* Control | *Aabr07025272.1* | -2.709187515 | 5.11E-07 | DOWN |
| Blood | Bone defect *vs.* Control | *Aabr07072184.1* | -9.344681127 | 5.27E-07 | DOWN |
| Blood | Bone defect *vs.* Control | *Tlr7* | -1.770149468 | 5.62E-07 | DOWN |
| Blood | Bone defect *vs.* Control | *Aabr07035776.1* | -1.926007678 | 7.77E-07 | DOWN |
| Blood | Bone defect *vs.* Control | *Dtx3l* | -1.766732094 | 8.58E-07 | DOWN |
| Blood | Bone defect *vs.* Control | *Klra22* | -3.296617447 | 8.92E-07 | DOWN |
| Blood | Bone defect *vs.* Control | *Aabr07006263.1* | -3.459318367 | 1.20E-06 | DOWN |
| Blood | Bone defect *vs.* Control | *Xcl1* | -3.416593787 | 1.76E-06 | DOWN |
| Blood | Bone defect *vs.* Control | *Aabr07051548.1* | -3.849066154 | 1.91E-06 | DOWN |
| Blood | Bone defect *vs.* Control | *Ms4a6a* | -2.772533834 | 2.11E-06 | DOWN |
| Blood | Bone defect *vs.* Control | *Igtp* | -1.971072372 | 2.18E-06 | DOWN |
| Blood | Bone defect *vs.* Control | *Rgd1565785* | -1.853143615 | 2.41E-06 | DOWN |
| Blood | Bone defect *vs.* Control | *Ac128859.3* | -1.895205243 | 2.48E-06 | DOWN |
| Blood | Bone defect *vs.* Control | *Ifi44l* | -2.212766459 | 2.47E-06 | DOWN |
| Blood | Bone defect *vs.* Control | *Aabr07068214.1* | -2.101982455 | 3.07E-06 | DOWN |
| Blood | Bone defect *vs.* Control | *Mnda* | -1.708291472 | 3.21E-06 | DOWN |
| Blood | Bone defect *vs.* Control | *Aabr07034362.2* | -2.251897809 | 3.28E-06 | DOWN |
| Blood | Bone defect *vs.* Control | *Aabr07065789.3* | -4.082114511 | 3.37E-06 | DOWN |
| Blood | Bone defect *vs.* Control | *Ms4a6bl* | -1.733343048 | 3.51E-06 | DOWN |
| Blood | Bone defect *vs.* Control | *Epsti1* | -1.67909794 | 3.72E-06 | DOWN |
| Blood | Bone defect *vs.* Control | *Prg2* | -3.082527351 | 4.24E-06 | DOWN |
| Blood | Bone defect *vs.* Control | *Irgm* | -2.028166563 | 4.54E-06 | DOWN |
| Blood | Bone defect *vs.* Control | *Pbk* | -3.603821884 | 4.61E-06 | DOWN |
| Blood | Bone defect *vs.* Control | *Ddx58* | -2.115440404 | 5.53E-06 | DOWN |
| Blood | Bone defect *vs.* Control | *Fbxo39* | -2.269978886 | 5.46E-06 | DOWN |
| Blood | Bone defect *vs.* Control | *L1cam* | 1.184357405 | 6.42E-06 | UP |
| Blood | Bone defect *vs.* Control | *Aabr07021221.1* | -1.519367 | 6.60E-06 | DOWN |
| Blood | Bone defect *vs.* Control | *Setdb2* | -2.169105102 | 6.82E-06 | DOWN |
| Blood | Bone defect *vs.* Control | *Ifi47* | -1.782403861 | 7.12E-06 | DOWN |
| Blood | Bone defect *vs.* Control | *Ddit3* | -1.786220179 | 7.31E-06 | DOWN |
| Blood | Bone defect *vs.* Control | *Jchain* | -2.152609562 | 7.49E-06 | DOWN |
| Blood | Bone defect *vs.* Control | *Myct1* | -1.944851031 | 7.66E-06 | DOWN |
| Blood | Bone defect *vs.* Control | *Ac118772.2* | -1.811804501 | 8.53E-06 | DOWN |
| Blood | Bone defect *vs.* Control | *Rt1-N3* | -1.970447351 | 9.37E-06 | DOWN |
| Blood | Bone defect *vs.* Control | *Nuf2* | -2.484019185 | 9.80E-06 | DOWN |
| Blood | Bone defect *vs.* Control | *Aabr07057347.1* | 1.631804099 | 1.00E-05 | UP |
| Blood | Bone defect *vs.* Control | *Pkr* | 1.866829268 | 1.03E-05 | UP |
| Blood | Bone defect *vs.* Control | *Sntb1* | -1.512855758 | 1.07E-05 | DOWN |
| Blood | Bone defect *vs.* Control | *Stmn1* | -1.660783412 | 1.08E-05 | DOWN |
| Blood | Bone defect *vs.* Control | *Ccna2* | -3.200579937 | 1.17E-05 | DOWN |
| Blood | Bone defect *vs.* Control | *Eomes* | -2.128894381 | 1.22E-05 | DOWN |
| Blood | Bone defect *vs.* Control | *Nos2* | -2.25501278 | 1.22E-05 | DOWN |
| Blood | Bone defect *vs.* Control | *Cxcl10* | -3.305449838 | 1.26E-05 | DOWN |
| Blood | Bone defect *vs.* Control | *Evc2* | -7.284489747 | 1.30E-05 | DOWN |
| Blood | Bone defect *vs.* Control | *Slc25a37* | 1.192043791 | 1.31E-05 | UP |
| Blood | Bone defect *vs.* Control | *Trex1* | -1.791567441 | 1.39E-05 | DOWN |
| Blood | Bone defect *vs.* Control | *Aabr07024203.1* | -2.200287971 | 1.48E-05 | DOWN |
| Blood | Bone defect *vs.* Control | *Loc100910979* | -1.665384896 | 1.54E-05 | DOWN |
| Blood | Bone defect *vs.* Control | *Ifi35* | -1.952646087 | 1.58E-05 | DOWN |
| Blood | Bone defect *vs.* Control | *Newgene_1308196* | -2.203084209 | 1.59E-05 | DOWN |
| Blood | Bone defect *vs.* Control | *Sp110* | -1.799853274 | 1.60E-05 | DOWN |
| Blood | Bone defect *vs.* Control | *Mcpt8l3* | -2.841446748 | 1.75E-05 | DOWN |
| Blood | Bone defect *vs.* Control | *Pclaf* | -3.018810863 | 1.83E-05 | DOWN |
| Blood | Bone defect *vs.* Control | *Cd300lf* | -2.50479537 | 2.10E-05 | DOWN |
| Blood | Bone defect *vs.* Control | *Parp14* | -1.558212099 | 2.14E-05 | DOWN |
| Blood | Bone defect *vs.* Control | *Mier3* | -1.590223487 | 2.26E-05 | DOWN |
| Blood | Bone defect *vs.* Control | *Rnase2* | 1.576216736 | 2.27E-05 | UP |
| Blood | Bone defect *vs.* Control | *Aurkb* | -1.907748558 | 2.68E-05 | DOWN |
| Blood | Bone defect *vs.* Control | *Ncaph* | -2.835926417 | 2.76E-05 | DOWN |
| Blood | Bone defect *vs.* Control | *Ifitm3* | -3.980491458 | 2.85E-05 | DOWN |
| Blood | Bone defect *vs.* Control | *Tbrg1* | -1.363607805 | 3.05E-05 | DOWN |
| Blood | Bone defect *vs.* Control | *Ms4a6e* | -1.363211295 | 3.23E-05 | DOWN |
| Blood | Bone defect *vs.* Control | *Sgk1* | -1.584415541 | 3.33E-05 | DOWN |
| Blood | Bone defect *vs.* Control | *Irf9* | -1.585951798 | 3.31E-05 | DOWN |
| Blood | Bone defect *vs.* Control | *Gbp2* | -1.60737486 | 3.28E-05 | DOWN |
| Blood | Bone defect *vs.* Control | *Gpr15* | -1.903161034 | 3.30E-05 | DOWN |
| Blood | Bone defect *vs.* Control | *Sap30* | -1.820013644 | 3.48E-05 | DOWN |
| Blood | Bone defect *vs.* Control | *Marcksl1* | -2.295341116 | 3.72E-05 | DOWN |
| Blood | Bone defect *vs.* Control | *Klrk1* | -1.495984546 | 4.26E-05 | DOWN |
| Blood | Bone defect *vs.* Control | *Ly6e* | -1.572178036 | 4.67E-05 | DOWN |
| Blood | Bone defect *vs.* Control | *Rgd1560925* | -2.171118322 | 4.77E-05 | DOWN |
| Blood | Bone defect *vs.* Control | *Cd7* | -1.636210158 | 4.90E-05 | DOWN |
| Blood | Bone defect *vs.* Control | *Loc102556085* | -2.06991707 | 5.31E-05 | DOWN |
| Blood | Bone defect *vs.* Control | *Ccl9* | -3.245105406 | 5.37E-05 | DOWN |
| Blood | Bone defect *vs.* Control | *Parp9* | -1.464166964 | 5.63E-05 | DOWN |
| Blood | Bone defect *vs.* Control | *Il18* | -2.572891716 | 5.66E-05 | DOWN |
| Blood | Bone defect *vs.* Control | *Kif11* | -1.88948865 | 5.90E-05 | DOWN |
| Blood | Bone defect *vs.* Control | *Aabr07060487.1* | -1.928227366 | 6.57E-05 | DOWN |
| Blood | Bone defect *vs.* Control | *Dsc2* | -2.493452157 | 6.81E-05 | DOWN |
| Blood | Bone defect *vs.* Control | *Rgd1561730* | 2.031175284 | 6.91E-05 | UP |
| Blood | Bone defect *vs.* Control | *Aabr07027872.1* | -1.560999905 | 6.99E-05 | DOWN |
| Blood | Bone defect *vs.* Control | *Serpinb10* | -1.554294831 | 7.38E-05 | DOWN |
| Blood | Bone defect *vs.* Control | *Ly49i4* | -2.303377697 | 7.45E-05 | DOWN |
| Blood | Bone defect *vs.* Control | *Erp27* | 2.427198354 | 7.69E-05 | UP |
| Blood | Bone defect *vs.* Control | *Oas1f* | -1.823720835 | 8.05E-05 | DOWN |
| Blood | Bone defect *vs.* Control | *Layn* | -1.866867258 | 8.12E-05 | DOWN |
| Blood | Bone defect *vs.* Control | *Cdk1* | -2.665309327 | 8.33E-05 | DOWN |
| Blood | Bone defect *vs.* Control | *Pml* | -1.447861825 | 8.40E-05 | DOWN |
| Blood | Bone defect *vs.* Control | *Ogfr* | -1.642053538 | 9.01E-05 | DOWN |
| Blood | Bone defect *vs.* Control | *Gbp6* | -1.714211758 | 9.17E-05 | DOWN |
| Blood | Bone defect *vs.* Control | *Plk1* | -2.675972017 | 9.16E-05 | DOWN |
| Blood | Bone defect *vs.* Control | *Tmem40* | -2.644421013 | 9.45E-05 | DOWN |
| Blood | Bone defect *vs.* Control | *Cep135* | -1.607850504 | 0.000102047 | DOWN |
| Blood | Bone defect *vs.* Control | *Loc100359515* | -2.156479831 | 0.00010287 | DOWN |
| Blood | Bone defect *vs.* Control | *Asf1b* | -2.240746693 | 0.000103765 | DOWN |
| Blood | Bone defect *vs.* Control | *Loc108348048* | -1.596490374 | 0.000115084 | DOWN |
| Blood | Bone defect *vs.* Control | *C1qtnf6* | -1.996632451 | 0.000115707 | DOWN |
| Blood | Bone defect *vs.* Control | *Gbp5* | -1.26997493 | 0.000114754 | DOWN |
| Blood | Bone defect *vs.* Control | *Isg20* | -1.862829039 | 0.00011395 | DOWN |
| Blood | Bone defect *vs.* Control | *Tapbp* | -1.444062649 | 0.000119397 | DOWN |
| Blood | Bone defect *vs.* Control | *Aabr07014290.1* | -1.359789862 | 0.00011981 | DOWN |
| Blood | Bone defect *vs.* Control | *Aabr07051548.2* | -4.371646132 | 0.00012095 | DOWN |
| Blood | Bone defect *vs.* Control | *Plac8* | -1.447759654 | 0.000134382 | DOWN |
| Blood | Bone defect *vs.* Control | *Cchcr1* | 1.049005387 | 0.000133975 | UP |
| Blood | Bone defect *vs.* Control | *Hspa1b* | -3.315287723 | 0.000136942 | DOWN |
| Blood | Bone defect *vs.* Control | *Plaat3* | -1.29363952 | 0.000142867 | DOWN |
| Blood | Bone defect *vs.* Control | *Gzmbl2* | -2.433539502 | 0.000145609 | DOWN |
| Blood | Bone defect *vs.* Control | *Rgs18* | -1.347078097 | 0.000149441 | DOWN |
| Blood | Bone defect *vs.* Control | *Trim21* | -1.643647049 | 0.000159257 | DOWN |
| Blood | Bone defect *vs.* Control | *Slc28a2* | -1.542476443 | 0.000163184 | DOWN |
| Blood | Bone defect *vs.* Control | *Klri1* | -2.086213392 | 0.000165369 | DOWN |
| Blood | Bone defect *vs.* Control | *Aabr07051718.1* | -4.658706723 | 0.000168368 | DOWN |
| Blood | Bone defect *vs.* Control | *Tmem140* | -1.433298671 | 0.000169446 | DOWN |
| Blood | Bone defect *vs.* Control | *Oma1* | -1.580334756 | 0.000176522 | DOWN |
| Blood | Bone defect *vs.* Control | *P2ry14* | -2.13615767 | 0.000181474 | DOWN |
| Blood | Bone defect *vs.* Control | *Cenph* | -2.527963216 | 0.000185115 | DOWN |
| Blood | Bone defect *vs.* Control | *Styk1* | -2.272112744 | 0.000188348 | DOWN |
| Blood | Bone defect *vs.* Control | *Aabr07052458.1* | -3.050840898 | 0.000189955 | DOWN |
| Blood | Bone defect *vs.* Control | *Mcpt8l2* | -2.745597726 | 0.000193666 | DOWN |
| Blood | Bone defect *vs.* Control | *Cenpa* | -2.277793248 | 0.000198123 | DOWN |
| Blood | Bone defect *vs.* Control | *Uhrf1* | -1.903225266 | 0.000213582 | DOWN |
| Blood | Bone defect *vs.* Control | *Loc100911104* | -2.102734894 | 0.000219942 | DOWN |
| Blood | Bone defect *vs.* Control | *Rt1-A1* | -1.269097269 | 0.000226195 | DOWN |
| Blood | Bone defect *vs.* Control | *Magohb* | -1.489088598 | 0.00023063 | DOWN |
| Blood | Bone defect *vs.* Control | *Rarres1* | -3.287052307 | 0.000232247 | DOWN |
| Blood | Bone defect *vs.* Control | *Stat1* | 1.389448473 | 0.000237044 | UP |
| Blood | Bone defect *vs.* Control | *Mrpl43* | -1.061955641 | 0.000236396 | DOWN |
| Blood | Bone defect *vs.* Control | *C1r* | -2.589973296 | 0.000241965 | DOWN |
| Blood | Bone defect *vs.* Control | *Olr1* | -1.551285685 | 0.00024556 | DOWN |
| Blood | Bone defect *vs.* Control | *Rrm2* | -1.71540061 | 0.000255365 | DOWN |
| Blood | Bone defect *vs.* Control | *Enpp4* | -1.482332638 | 0.000257311 | DOWN |
| Blood | Bone defect *vs.* Control | *Trafd1* | -1.541624401 | 0.000260286 | DOWN |
| Blood | Bone defect *vs.* Control | *Rt1-A2* | -1.391741605 | 0.000262534 | DOWN |
| Blood | Bone defect *vs.* Control | *Mzb1* | -1.670300504 | 0.000267414 | DOWN |
| Blood | Bone defect *vs.* Control | *Tent5a* | -1.512525248 | 0.000272029 | DOWN |
| Blood | Bone defect *vs.* Control | *Loc100359539* | -1.741640396 | 0.000275462 | DOWN |
| Blood | Bone defect *vs.* Control | *Cadm1* | -2.175874797 | 0.000275141 | DOWN |
| Blood | Bone defect *vs.* Control | *Defb14* | -2.919475822 | 0.000277652 | DOWN |
| Blood | Bone defect *vs.* Control | *Cdc20* | -1.502653849 | 0.000280132 | DOWN |
| Blood | Bone defect *vs.* Control | *Aabr07021804.1* | -1.996694896 | 0.000283143 | DOWN |
| Blood | Bone defect *vs.* Control | *Loc691670* | -2.942783937 | 0.000287721 | DOWN |
| Blood | Bone defect *vs.* Control | *Ncr1* | -1.655249794 | 0.000289602 | DOWN |
| Blood | Bone defect *vs.* Control | *Adar* | -1.298727678 | 0.0002956 | DOWN |
| Blood | Bone defect *vs.* Control | *Loc100910650* | -1.363963776 | 0.000316208 | DOWN |
| Blood | Bone defect *vs.* Control | *Rt1-S3* | -1.430363042 | 0.000320861 | DOWN |
| Blood | Bone defect *vs.* Control | *Plekhb1* | 2.133259578 | 0.000319533 | UP |
| Blood | Bone defect *vs.* Control | *Loc688932* | -1.389503634 | 0.000321046 | DOWN |
| Blood | Bone defect *vs.* Control | *Loc690000* | -1.460557408 | 0.000345656 | DOWN |
| Blood | Bone defect *vs.* Control | *Mcm5* | -1.378334839 | 0.000359041 | DOWN |
| Blood | Bone defect *vs.* Control | *Loc690045* | -1.900793111 | 0.000365434 | DOWN |
| Blood | Bone defect *vs.* Control | *Rt1-T24-4* | -2.258810861 | 0.000381967 | DOWN |
| Blood | Bone defect *vs.* Control | *Ngp* | 1.469646083 | 0.000391167 | UP |
| Blood | Bone defect *vs.* Control | *Gpr55* | -2.984074281 | 0.000401413 | DOWN |
| Blood | Bone defect *vs.* Control | *Aabr07001389.1* | -1.544250402 | 0.000402902 | DOWN |
| Blood | Bone defect *vs.* Control | *Nampt* | -1.397146928 | 0.000410235 | DOWN |
| Blood | Bone defect *vs.* Control | *Apobec1* | -1.733408431 | 0.000412359 | DOWN |
| Blood | Bone defect *vs.* Control | *Mgc108823* | -1.42683685 | 0.000409687 | DOWN |
| Blood | Bone defect *vs.* Control | *Sp100* | -1.424986198 | 0.00042532 | DOWN |
| Blood | Bone defect *vs.* Control | *Ckap2* | -1.708759373 | 0.000425948 | DOWN |
| Blood | Bone defect *vs.* Control | *Mitd1* | -1.474299073 | 0.000437559 | DOWN |
| Blood | Bone defect *vs.* Control | *Rhd* | 1.29980633 | 0.000452611 | UP |
| Blood | Bone defect *vs.* Control | *Ccr1l1* | -2.145735117 | 0.000484691 | DOWN |
| Blood | Bone defect *vs.* Control | *Ca11* | 1.183791339 | 0.000488209 | UP |
| Blood | Bone defect *vs.* Control | *Tapbpl* | -1.201814627 | 0.000488788 | DOWN |
| Blood | Bone defect *vs.* Control | *Loc685067* | -1.174181966 | 0.000496227 | DOWN |
| Blood | Bone defect *vs.* Control | *Rt1-Ce4* | -1.187949184 | 0.000509663 | DOWN |
| Blood | Bone defect *vs.* Control | *Sowaha* | 1.590138604 | 0.000514673 | UP |
| Blood | Bone defect *vs.* Control | *Lag3* | -1.642463252 | 0.000518733 | DOWN |
| Blood | Bone defect *vs.* Control | *Hist1h2bd* | 1.416607233 | 0.000542101 | UP |
| Blood | Bone defect *vs.* Control | *Slc35d1* | -1.75330894 | 0.00055126 | DOWN |
| Blood | Bone defect *vs.* Control | *Loc108348155* | 1.419694468 | 0.000610146 | UP |
| Blood | Bone defect *vs.* Control | *B2m* | -1.333148039 | 0.000621122 | DOWN |
| Blood | Bone defect *vs.* Control | *Sdc1* | -1.588197179 | 0.000632294 | DOWN |
| Blood | Bone defect *vs.* Control | *Cd300e* | -1.824043622 | 0.000651232 | DOWN |
| Blood | Bone defect *vs.* Control | *Ly6al* | -3.398452007 | 0.000664446 | DOWN |
| Blood | Bone defect *vs.* Control | *Aabr07030796.1* | -2.423733866 | 0.00067831 | DOWN |
| Blood | Bone defect *vs.* Control | *Scimp* | -1.719876945 | 0.000704598 | DOWN |
| Blood | Bone defect *vs.* Control | *Gbp4* | -1.454500685 | 0.000717547 | DOWN |
| Blood | Bone defect *vs.* Control | *Rab37* | -1.229160054 | 0.000717862 | DOWN |
| Blood | Bone defect *vs.* Control | *Srgap2* | -1.316331565 | 0.000754938 | DOWN |
| Blood | Bone defect *vs.* Control | *Helz2* | -1.275930016 | 0.000751839 | DOWN |
| Blood | Bone defect *vs.* Control | *Ppef1* | -3.766703659 | 0.000748334 | DOWN |
| Blood | Bone defect *vs.* Control | *Ndc80* | -1.478511947 | 0.00076653 | DOWN |
| Blood | Bone defect *vs.* Control | *Fbxo5* | -1.850821398 | 0.000791719 | DOWN |
| Blood | Bone defect *vs.* Control | *Fpr2* | 2.703566334 | 0.000794887 | UP |
| Blood | Bone defect *vs.* Control | *Adgrg5* | -1.5262406 | 0.000798564 | DOWN |
| Blood | Bone defect *vs.* Control | *Loc100911515* | -1.185534715 | 0.000814522 | DOWN |
| Blood | Bone defect *vs.* Control | *Psme1* | -1.206160812 | 0.000844397 | DOWN |
| Blood | Bone defect *vs.* Control | *Ncoa4* | 1.051263992 | 0.000855879 | UP |
| Blood | Bone defect *vs.* Control | *Cyp27a1* | -2.195312778 | 0.000865499 | DOWN |
| Blood | Bone defect *vs.* Control | *Il12rb2* | -1.656161691 | 0.000886027 | DOWN |
| Blood | Bone defect *vs.* Control | *Hsph1* | -1.427612356 | 0.000901156 | DOWN |
| Blood | Bone defect *vs.* Control | *Kif22* | -1.935958852 | 0.000901438 | DOWN |
| Blood | Bone defect *vs.* Control | *Hist1h2af* | 1.397791551 | 0.000928623 | UP |
| Blood | Bone defect *vs.* Control | *Aabr07051592.1* | 1.646443773 | 0.000944272 | UP |
| Blood | Bone defect *vs.* Control | *Ccr5* | -1.38014716 | 0.000973767 | DOWN |
| Blood | Bone defect *vs.* Control | *Aabr07061001.1* | -1.893099408 | 0.000994789 | DOWN |
| Blood | Bone defect *vs.* Control | *Psme2* | -1.087683234 | 0.001007652 | DOWN |
| Blood | Bone defect *vs.* Control | *Hepacam2* | 1.125289642 | 0.00102525 | UP |
| Blood | Bone defect *vs.* Control | *Stfa2* | -1.318144727 | 0.001045662 | DOWN |
| Blood | Bone defect *vs.* Control | *B4galt5* | -1.39608788 | 0.00108824 | DOWN |
| Blood | Bone defect *vs.* Control | *Rgd1309362* | -1.366281007 | 0.001088472 | DOWN |
| Blood | Bone defect *vs.* Control | *Pttg1* | 1.120028788 | 0.001110732 | UP |
| Blood | Bone defect *vs.* Control | *Aldh7a1* | -1.7240981 | 0.001109832 | DOWN |
| Bone | Bone defect *vs.* Control | *Aabr07070238.3* | -9.526926469 | 9.43E-06 | DOWN |
| Bone | Bone defect *vs.* Control | *Aabr07060963.3* | 3.898025617 | 9.61E-06 | UP |
| Bone | Bone defect *vs.* Control | *Ac098750.1* | -8.651199314 | 1.55E-05 | DOWN |
| Bone | Bone defect *vs.* Control | *Aabr07053866.1* | -7.196373407 | 3.18E-05 | DOWN |
| Bone | Bone defect *vs.* Control | *Loc100362751* | -4.470832024 | 5.08E-05 | DOWN |
| Bone | Bone defect *vs.* Control | *Rt1-Ce5* | -1.958342136 | 6.67E-05 | DOWN |
| Bone | Bone defect *vs.* Control | *Loc100911575* | -4.085392701 | 6.80E-05 | DOWN |
| Bone | Bone defect *vs.* Control | *Aabr07061072.1* | 2.480745784 | 7.81E-05 | UP |
| Bone | Bone defect *vs.* Control | *Slfn5* | -1.64781578 | 0.000101526 | DOWN |
| Bone | Bone defect *vs.* Control | *Sct* | -3.878194481 | 0.000126123 | DOWN |
| Bone | Bone defect *vs.* Control | *Loc103689965* | -1.517446951 | 0.00013211 | DOWN |
| Bone | Bone defect *vs.* Control | *Rplp2* | -4.28017699 | 0.000148337 | DOWN |
| Bone | Bone defect *vs.* Control | *Rgd1564613* | -3.761799407 | 0.000163641 | DOWN |
| Bone | Bone defect *vs.* Control | *Fam210a* | -1.953329766 | 0.000151692 | DOWN |
| Bone | Bone defect *vs.* Control | *Catsper2* | -3.138234018 | 0.000159518 | DOWN |
| Bone | Bone defect *vs.* Control | *Trim30c* | -1.838283997 | 0.000161954 | DOWN |
| Bone | Bone defect *vs.* Control | *Panx3* | 2.60125264 | 0.000140648 | UP |
| Bone | Bone defect *vs.* Control | *Gp1bb* | -2.048498727 | 0.000159624 | DOWN |
| Bone | Bone defect *vs.* Control | *Anp32a* | -3.461943411 | 0.00015232 | DOWN |
| Bone | Bone defect *vs.* Control | *Ac134224.3* | -4.400743974 | 0.000146982 | DOWN |
| Blood | Osteoking *vs.* Bone defect | *Aabr07015078.1* | 3.05003789 | 2.94E-14 | UP |
| Blood | Osteoking *vs.* Bone defect | *Aabr07015066.1* | 3.300029785 | 1.46E-13 | UP |
| Blood | Osteoking *vs.* Bone defect | *Aabr07063424.1* | 2.797386361 | 7.64E-13 | UP |
| Blood | Osteoking *vs.* Bone defect | *Loc102549714* | 1.670260562 | 2.12E-11 | UP |
| Blood | Osteoking *vs.* Bone defect | *Aabr07026058.1* | -1.643868391 | 2.78E-10 | DOWN |
| Blood | Osteoking *vs.* Bone defect | *Plekhb1* | -2.857360533 | 6.87E-10 | DOWN |
| Blood | Osteoking *vs.* Bone defect | *Aabr07015055.1* | 2.770411071 | 1.80E-09 | UP |
| Blood | Osteoking *vs.* Bone defect | *Selenbp1* | 1.057756732 | 4.54E-09 | UP |
| Blood | Osteoking *vs.* Bone defect | *Ac113771.1* | -1.781316925 | 5.19E-08 | DOWN |
| Blood | Osteoking *vs.* Bone defect | *Loc108352861* | -1.54905271 | 7.38E-08 | DOWN |
| Blood | Osteoking *vs.* Bone defect | *Evc2* | 7.094128533 | 1.39E-07 | UP |
| Blood | Osteoking *vs.* Bone defect | *Aabr07072078.1* | -1.533035076 | 1.39E-07 | DOWN |
| Blood | Osteoking *vs.* Bone defect | *Aabr07064000.1* | -1.46114285 | 1.43E-07 | DOWN |
| Blood | Osteoking *vs.* Bone defect | *Aabr07027722.1* | 1.351151301 | 1.36E-07 | UP |
| Blood | Osteoking *vs.* Bone defect | *Atp6v1f* | -1.289606216 | 3.19E-07 | DOWN |
| Blood | Osteoking *vs.* Bone defect | *Aabr07015056.1* | 4.591831072 | 3.23E-07 | UP |
| Blood | Osteoking *vs.* Bone defect | *Aabr07049516.1* | -1.377404842 | 5.02E-07 | DOWN |
| Blood | Osteoking *vs.* Bone defect | *Aabr07064810.1* | -1.351015239 | 1.09E-06 | DOWN |
| Blood | Osteoking *vs.* Bone defect | *Aabr07068536.1* | -1.393798527 | 1.24E-06 | DOWN |
| Blood | Osteoking *vs.* Bone defect | *Rgd1564606* | -1.105571071 | 6.65E-06 | DOWN |
| Blood | Osteoking *vs.* Bone defect | *Aabr07051658.1* | 2.443834259 | 6.44E-06 | UP |
| Blood | Osteoking *vs.* Bone defect | *Aabr07034315.1* | -1.372873234 | 7.36E-06 | DOWN |
| Blood | Osteoking *vs.* Bone defect | *Aabr07028839.1* | -1.131771686 | 7.81E-06 | DOWN |
| Blood | Osteoking *vs.* Bone defect | *Aabr07072207.1* | -1.06669095 | 8.62E-06 | DOWN |
| Blood | Osteoking *vs.* Bone defect | *Aabr07019341.1* | -1.540616698 | 9.93E-06 | DOWN |
| Blood | Osteoking *vs.* Bone defect | *Aabr07048013.1* | -1.50240494 | 1.05E-05 | DOWN |
| Blood | Osteoking *vs.* Bone defect | *Aabr07015080.2* | 2.342582948 | 1.08E-05 | UP |
| Blood | Osteoking *vs.* Bone defect | *Milr1* | -1.257078444 | 1.49E-05 | DOWN |
| Blood | Osteoking *vs.* Bone defect | *Loc691427* | -1.274132119 | 1.61E-05 | DOWN |
| Blood | Osteoking *vs.* Bone defect | *Aabr07027854.1* | -1.175206324 | 1.62E-05 | DOWN |
| Blood | Osteoking *vs.* Bone defect | *Aabr07026317.1* | -1.288151429 | 1.69E-05 | DOWN |
| Blood | Osteoking *vs.* Bone defect | *Ac099453.1* | -1.383976639 | 2.20E-05 | DOWN |
| Blood | Osteoking *vs.* Bone defect | *Prok2* | -1.33990462 | 2.37E-05 | DOWN |
| Blood | Osteoking *vs.* Bone defect | *Aabr07048031.1* | -1.347203553 | 2.48E-05 | DOWN |
| Blood | Osteoking *vs.* Bone defect | *Aabr07019663.1* | -1.553995668 | 2.62E-05 | DOWN |
| Blood | Osteoking *vs.* Bone defect | *Aabr07027447.1* | -2.007289496 | 2.75E-05 | DOWN |
| Blood | Osteoking *vs.* Bone defect | *Ac135409.1* | -1.548684107 | 2.95E-05 | DOWN |
| Blood | Osteoking *vs.* Bone defect | *Aabr07024907.1* | -1.730345459 | 2.97E-05 | DOWN |
| Blood | Osteoking *vs.* Bone defect | *Selenow* | -1.537792118 | 4.97E-05 | DOWN |
| Blood | Osteoking *vs.* Bone defect | *Mrpl33* | -1.216026935 | 5.85E-05 | DOWN |
| Blood | Osteoking *vs.* Bone defect | *Loc100365810* | -1.18269268 | 6.20E-05 | DOWN |
| Blood | Osteoking *vs.* Bone defect | *Loc690468* | -1.560816937 | 6.88E-05 | DOWN |
| Blood | Osteoking *vs.* Bone defect | *Aabr07040944.1* | -3.018109298 | 7.65E-05 | DOWN |
| Blood | Osteoking *vs.* Bone defect | *Aabr07031089.1* | -1.040293108 | 7.60E-05 | DOWN |
| Blood | Osteoking *vs.* Bone defect | *Rps29* | -1.18202933 | 7.82E-05 | DOWN |
| Blood | Osteoking *vs.* Bone defect | *Aabr07059198.1* | -1.244544183 | 8.53E-05 | DOWN |
| Blood | Osteoking *vs.* Bone defect | *Ac095947.3* | -1.349885789 | 9.77E-05 | DOWN |
| Blood | Osteoking *vs.* Bone defect | *Aabr07072400.1* | -1.111841174 | 0.000111373 | DOWN |
| Blood | Osteoking *vs.* Bone defect | *Atp5me* | -1.211025319 | 0.000118568 | DOWN |
| Blood | Osteoking *vs.* Bone defect | *Loc497796* | 1.009756277 | 0.000138334 | UP |
| Blood | Osteoking *vs.* Bone defect | *Loc691716* | -1.202318143 | 0.000156781 | DOWN |
| Blood | Osteoking *vs.* Bone defect | *Loc100362339* | -1.038528221 | 0.000216888 | DOWN |
| Blood | Osteoking *vs.* Bone defect | *Loc103694169* | -1.293820954 | 0.000223349 | DOWN |
| Blood | Osteoking *vs.* Bone defect | *Loc108348144* | -1.13107117 | 0.00022161 | DOWN |
| Blood | Osteoking *vs.* Bone defect | *Aabr07053749.2* | -1.266554211 | 0.000224599 | DOWN |
| Blood | Osteoking *vs.* Bone defect | *Pkr* | -1.429428134 | 0.003541885 | DOWN |
| Blood | Osteoking *vs.* Bone defect | *Mlkl* | -1.145052192 | 0.005774193 | DOWN |
| Blood | Osteoking *vs.* Bone defect | *Stat1* | -1.320290297 | 0.006941121 | DOWN |
| Blood | Osteoking *vs.* Bone defect | *Zbp1* | -1.280626422 | 0.009695681 | DOWN |
| Bone | Osteoking *vs.* Bone defect | *Gp1bb* | 5.781848633 | 2.55E-13 | UP |
| Bone | Osteoking *vs.* Bone defect | *Pla2g4c* | -3.795274555 | 6.90E-10 | DOWN |
| Bone | Osteoking *vs.* Bone defect | *Ac117058.1* | 5.169811535 | 5.87E-09 | UP |
| Bone | Osteoking *vs.* Bone defect | *Il5ra* | -2.583273064 | 5.89E-06 | DOWN |
| Bone | Osteoking *vs.* Bone defect | *Scand1* | 1.822690887 | 9.15E-06 | UP |
| Bone | Osteoking *vs.* Bone defect | *Ac127784.5* | 2.213804852 | 2.26E-05 | UP |

**Table S4 The differentially expressed genes (DEGs) with both functional interactions and significant co-expression correlations.**

| **Node1**  **(Gene symbol)** | **Node2**  **(Gene symbol)** | **Combined score** | **Pearson correlation coefficients in whole blood** | **Pearson correlation coefficients in bone tissue** |
| --- | --- | --- | --- | --- |
| *Adar* | *Oas2* | 0.718 | 0.982706643 | 0.95058581 |
| *Adar* | *Oas3* | 0.782 | 0.976498869 | 0.96109565 |
| *Asf1b* | *Cdc20* | 0.839 | 0.806402967 | 0.873673319 |
| *Aurkb* | *Cenpa* | 0.926 | 0.98444602 | 0.918219392 |
| *Aurkb* | *Kif11* | 0.982 | 0.973733503 | 0.913961159 |
| *Aurkb* | *Kif22* | 0.843 | 0.961780522 | -0.866140182 |
| *Aurkb* | *LOC100359539* | 0.717 | 0.971971468 | 0.835795266 |
| *Aurkb* | *Mcm5* | 0.799 | 0.977142359 | 0.861165134 |
| *Aurkb* | *Ncaph* | 0.967 | 0.97278563 | 0.885592358 |
| *Aurkb* | *Ndc80* | 0.993 | 0.973301838 | 0.863355839 |
| *Aurkb* | *Rrm2* | 0.789 | 0.985731432 | 0.940456022 |
| *Aurkb* | *Stmn1* | 0.813 | 0.936252003 | -0.963472331 |
| *Aurkb* | *Uhrf1* | 0.775 | 0.958246155 | 0.932163596 |
| *Aurkb* | *Ccna2* | 0.983 | 0.963367494 | 0.902773066 |
| *Aurkb* | *Cdc20* | 0.995 | 0.858272238 | 0.906024244 |
| *B2m* | *Klrk1* | 0.872 | 0.87961251 | 0.856643198 |
| *B2m* | *RT1-A1* | 0.913 | 0.966728698 | 0.715554286 |
| *B2m* | *RT1-N3* | 0.901 | 0.948283901 | 0.963627919 |
| *B2m* | *RT1-S3* | 0.924 | 0.960802347 | 0.974625395 |
| *Bst2* | *Isg15* | 0.796 | 0.753403576 | 0.899689107 |
| *C1r* | *C2* | 0.852 | 0.970578721 | 0.945817292 |
| *C1r* | *LOC103689965* | 0.948 | 0.898129562 | 0.849602604 |
| *C2* | *LOC103689965* | 0.918 | 0.904432316 | 0.960678432 |
| *Ccna2* | *Cdc20* | 0.999 | 0.878464512 | 0.932259302 |
| *Ccna2* | *Cdk1* | 0.999 | 0.945578938 | 0.743419049 |
| *Ccna2* | *Cenpa* | 0.716 | 0.946146583 | 0.865567763 |
| *Ccna2* | *Kif11* | 0.976 | 0.972214501 | 0.917489613 |
| *Ccna2* | *LOC100359539* | 0.778 | 0.941791834 | 0.924420499 |
| *Ccna2* | *Mcm5* | 0.926 | 0.986318356 | 0.930216701 |
| *Ccna2* | *Ncaph* | 0.843 | 0.988669123 | 0.957321319 |
| *Ccna2* | *Ndc80* | 0.938 | 0.914784291 | 0.872744738 |
| *Ccna2* | *Rrm2* | 0.831 | 0.929485462 | 0.905358415 |
| *Ccna2* | *Stmn1* | 0.768 | 0.970389502 | -0.955149076 |
| *Ccna2* | *Uhrf1* | 0.818 | 0.971866976 | 0.942924127 |
| *Cdc20* | *Cdk1* | 0.999 | 0.86852542 | 0.739823921 |
| *Cdc20* | *Cenpa* | 0.802 | 0.810847709 | 0.958437502 |
| *Cdc20* | *Ckap2* | 0.716 | 0.81560227 | 0.928194485 |
| *Cdc20* | *Kif11* | 0.98 | 0.852176158 | 0.879108044 |
| *Cdc20* | *Kif22* | 0.728 | 0.861931534 | -0.832866413 |
| *Cdc20* | *LOC100359539* | 0.836 | 0.930597113 | 0.86010745 |
| *Cdc20* | *Mcm5* | 0.96 | 0.891307795 | 0.95145082 |
| *Cdc20* | *Ncaph* | 0.827 | 0.869806307 | 0.883454981 |
| *Cdc20* | *Ndc80* | 0.972 | 0.818241443 | 0.799232239 |
| *Cdc20* | *Rrm2* | 0.832 | 0.867268075 | 0.91119079 |
| *Cdc20* | *Uhrf1* | 0.85 | 0.940623568 | 0.902441543 |
| *Cdk1* | *Cenpa* | 0.735 | 0.979136178 | 0.743902012 |
| *Cdk1* | *Ckap2* | 0.729 | 0.986984346 | 0.839919159 |
| *Cdk1* | *LOC100359539* | 0.826 | 0.968703444 | 0.760174229 |
| *Cdk1* | *Mcm5* | 0.971 | 0.966624326 | 0.819808789 |
| *Cdk1* | *Ncaph* | 0.769 | 0.963656784 | 0.765811497 |
| *Cdk1* | *Pbk* | 0.892 | 0.916696929 | 0.816904347 |
| *Cenpa* | *Kif11* | 0.748 | 0.950578741 | 0.837460457 |
| *Cenpa* | *Ndc80* | 0.794 | 0.969900394 | 0.77731719 |
| *Cxcl10* | *Isg15* | 0.806 | 0.864611068 | 0.954972002 |
| *Cxcl10* | *Mx2* | 0.742 | 0.96483313 | 0.918535787 |
| *Ddx58* | *Eif2ak2* | 0.743 | 0.979548833 | 0.977548962 |
| *Ddx58* | *Herc6* | 0.769 | 0.936434682 | 0.913231312 |
| *Ddx58* | *Ifi44* | 0.789 | 0.974441948 | 0.836454547 |
| *Ddx58* | *Ifi44l* | 0.749 | 0.961802115 | 0.881897243 |
| *Ddx58* | *Ifih1* | 0.745 | 0.992359933 | 0.966526908 |
| *Ddx58* | *Ifit2* | 0.856 | 0.994855617 | 0.986897167 |
| *Ddx58* | *Ifit3* | 0.87 | 0.988946459 | 0.962885062 |
| *Ddx58* | *Irf7* | 0.908 | 0.839722727 | 0.939988079 |
| *Ddx58* | *Irf9* | 0.764 | 0.938256181 | 0.875987445 |
| *Ddx58* | *Mx1* | 0.857 | 0.990120782 | 0.978684696 |
| *Ddx58* | *Oas1a* | 0.767 | 0.910081959 | 0.976398753 |
| *Ddx58* | *Oasl* | 0.903 | 0.918018545 | 0.940929618 |
| *Ddx58* | *Parp9* | 0.859 | 0.963412533 | 0.954199975 |
| *Ddx58* | *Rtp4* | 0.748 | 0.954086672 | 0.95786545 |
| *Ddx58* | *Stat1* | 0.832 | 0.964594367 | 0.942626699 |
| *Ddx58* | *Usp18* | 0.832 | 0.969363643 | 0.97417152 |
| *Dhx58* | *Ifit3* | 0.746 | 0.982963767 | 0.764791595 |
| *Dhx58* | *Isg15* | 0.913 | 0.846092347 | 0.979075572 |
| *Dhx58* | *Mx2* | 0.811 | 0.977721458 | 0.845539339 |
| *Dhx58* | *Oasl2* | 0.77 | 0.973360195 | 0.922064929 |
| *Dhx58* | *Parp9* | 0.717 | 0.960663173 | 0.786268963 |
| *Dhx58* | *Rtp4* | 0.747 | 0.99910241 | 0.765856314 |
| *Dtx3l* | *Ifih1* | 0.775 | 0.976755311 | 0.994141674 |
| *Dtx3l* | *Parp14* | 0.918 | 0.995409487 | 0.989623385 |
| *Dtx3l* | *Parp9* | 0.997 | 0.990092961 | 0.934424504 |
| *Dtx3l* | *Usp18* | 0.721 | 0.913865553 | 0.948223348 |
| *Eif2ak2* | *Herc6* | 0.704 | 0.882611083 | 0.973532269 |
| *Eif2ak2* | *Ifi44* | 0.718 | 0.975791818 | 0.895574569 |
| *Eif2ak2* | *Ifit2* | 0.751 | 0.965711752 | 0.98356847 |
| *Eif2ak2* | *Ifit3* | 0.829 | 0.984134622 | 0.958812392 |
| *Eif2ak2* | *Irf7* | 0.73 | 0.828960173 | 0.981757045 |
| *Eif2ak2* | *Mx1* | 0.802 | 0.993185313 | 0.978743306 |
| *Eif2ak2* | *Oas2* | 0.731 | 0.973064074 | 0.955906857 |
| *Eif2ak2* | *Parp14* | 0.774 | 0.988556711 | 0.988272116 |
| *Eif2ak2* | *Stat1* | 0.877 | 0.983791871 | 0.940960869 |
| *Eif2ak2* | *Usp18* | 0.974 | 0.947003534 | 0.982684113 |
| *Epsti1* | *Mx1* | 0.727 | 0.97045933 | 0.78541747 |
| *Gbp2* | *Ifit2* | 0.737 | 0.93618148 | 0.971935788 |
| *Gbp2* | *Igtp* | 0.775 | 0.942151448 | 0.979832755 |
| *Gbp2* | *Irf7* | 0.735 | 0.899238279 | 0.931995854 |
| *Gbp2* | *Irgm* | 0.839 | 0.985124937 | 0.970156761 |
| *Gbp2* | *Mx1* | 0.794 | 0.978734327 | 0.949888232 |
| *Gbp2* | *Parp14* | 0.71 | 0.953390761 | 0.972288673 |
| *Gbp2* | *Parp9* | 0.713 | 0.980141118 | 0.920717157 |
| *Gbp2* | *Stat1* | 0.872 | 0.996019124 | 0.990004898 |
| *Gbp4* | *Ifi47* | 0.716 | 0.971977048 | 0.898642162 |
| *Gbp4* | *Ifit2* | 0.753 | 0.903785439 | 0.894001507 |
| *Gbp4* | *Ifit3* | 0.771 | 0.948255078 | 0.794280043 |
| *Gzmb* | *Gzmbl2* | 0.814 | 0.990743674 | 0.931349855 |
| *Helz2* | *Parp14* | 0.811 | 0.99133548 | 0.971265682 |
| *Herc6* | *Ifi44* | 0.757 | 0.949011835 | 0.889614162 |
| *Herc6* | *Ifi44l* | 0.77 | 0.966445083 | 0.962674473 |
| *Herc6* | *Ifih1* | 0.732 | 0.929474191 | 0.927124608 |
| *Herc6* | *Ifit2* | 0.807 | 0.947400004 | 0.938734351 |
| *Herc6* | *Ifit3* | 0.851 | 0.945226462 | 0.905805457 |
| *Herc6* | *Irf7* | 0.75 | 0.933926926 | 0.993965937 |
| *Herc6* | *Mx1* | 0.901 | 0.922901867 | 0.953410067 |
| *Herc6* | *Oas1a* | 0.737 | 0.965505033 | 0.961355458 |
| *Herc6* | *Oasl* | 0.738 | 0.979342679 | 0.994365659 |
| *Herc6* | *Parp12* | 0.707 | 0.943733946 | 0.966431695 |
| *Herc6* | *Parp9* | 0.748 | 0.856222073 | 0.962441256 |
| *Herc6* | *Stat1* | 0.703 | 0.906964228 | 0.899985496 |
| *Herc6* | *Usp18* | 0.872 | 0.98293934 | 0.957173772 |
| *Ifi27* | *Mx2* | 0.815 | 0.909194602 | 0.961469553 |
| *Ifi27* | *Isg15* | 0.873 | 0.860840475 | 0.993267234 |
| *Ifi35* | *Ifi44* | 0.814 | 0.931391942 | 0.801898002 |
| *Ifi35* | *Ifi44l* | 0.778 | 0.956675272 | 0.707837431 |
| *Ifi35* | *Ifit3* | 0.862 | 0.892348565 | 0.747684308 |
| *Ifi35* | *Isg15* | 0.798 | 0.940964862 | 0.835524363 |
| *Ifi35* | *Mx2* | 0.769 | 0.893793088 | 0.701145021 |
| *Ifi35* | *Psme2* | 0.762 | 0.812459896 | 0.762131547 |
| *Ifi35* | *Ube2l6* | 0.815 | 0.973700272 | 0.709238888 |
| *Ifi44* | *Ifih1* | 0.844 | 0.980953683 | 0.755925818 |
| *Ifi44* | *Ifit2* | 0.868 | 0.966046244 | 0.821696462 |
| *Ifi44* | *Ifit3* | 0.881 | 0.989589719 | 0.921809899 |
| *Ifi44* | *Irf7* | 0.852 | 0.926880945 | 0.89064548 |
| *Ifi44* | *Isg15* | 0.863 | 0.835459065 | 0.848497053 |
| *Ifi44* | *Mx1* | 0.787 | 0.989157644 | 0.852277414 |
| *Ifi44* | *Oas1a* | 0.725 | 0.970835814 | 0.87132886 |
| *Ifi44* | *Oas2* | 0.812 | 0.97137936 | 0.871173351 |
| *Ifi44* | *Oas3* | 0.72 | 0.988327451 | 0.813202152 |
| *Ifi44* | *Oasl* | 0.813 | 0.968419023 | 0.868432426 |
| *Ifi44* | *Oasl2* | 0.774 | 0.983154508 | 0.725974811 |
| *Ifi44* | *Parp14* | 0.759 | 0.944566977 | 0.82602864 |
| *Ifi44* | *Parp9* | 0.702 | 0.96793934 | 0.949190852 |
| *Ifi44* | *Rtp4* | 0.809 | 0.995901121 | 0.941445633 |
| *Ifi44* | *Stat1* | 0.77 | 0.987451257 | 0.704187591 |
| *Ifi44* | *Usp18* | 0.846 | 0.989268197 | 0.900815007 |
| *Ifi44l* | *Ifih1* | 0.792 | 0.964580729 | 0.848465326 |
| *Ifi44l* | *Ifit2* | 0.809 | 0.95407706 | 0.887379207 |
| *Ifi44l* | *Ifit3* | 0.855 | 0.979025629 | 0.92186256 |
| *Ifi44l* | *Irf7* | 0.765 | 0.952796236 | 0.957529201 |
| *Ifi44l* | *Isg15* | 0.852 | 0.879625149 | 0.707042356 |
| *Ifi44l* | *Mx1* | 0.855 | 0.974070485 | 0.909160732 |
| *Ifi44l* | *Oasl* | 0.813 | 0.985356819 | 0.947094534 |
| *Ifi44l* | *Parp9* | 0.712 | 0.942709062 | 0.974531573 |
| *Ifi44l* | *Rtp4* | 0.733 | 0.996320102 | 0.961871481 |
| *Ifi44l* | *Stat1* | 0.748 | 0.976307613 | 0.816009744 |
| *Ifi44l* | *Usp18* | 0.721 | 0.993653522 | 0.939250788 |
| *Ifi47* | *Ifit2* | 0.756 | 0.965183554 | 0.971746351 |
| *Ifi47* | *Ifit3* | 0.773 | 0.992470342 | 0.90009733 |
| *Ifi47* | *Irf7* | 0.72 | 0.910850177 | 0.948737405 |
| *Ifi47* | *Parp14* | 0.732 | 0.955222423 | 0.980364131 |
| *Ifi47* | *Stat1* | 0.731 | 0.995249231 | 0.956704133 |
| *Ifih1* | *Ifit2* | 0.884 | 0.989995387 | 0.988379681 |
| *Ifih1* | *Ifit3* | 0.872 | 0.996571745 | 0.893601483 |
| *Ifih1* | *Irf7* | 0.901 | 0.850953315 | 0.937599211 |
| *Ifih1* | *Mx1* | 0.87 | 0.994855539 | 0.95701919 |
| *Ifih1* | *Oas2* | 0.717 | 0.948577788 | 0.909817169 |
| *Ifih1* | *Oasl* | 0.838 | 0.9242415 | 0.952036816 |
| *Ifih1* | *Parp12* | 0.784 | 0.987536302 | 0.981628139 |
| *Ifih1* | *Parp14* | 0.852 | 0.966976578 | 0.987056075 |
| *Ifih1* | *Parp9* | 0.878 | 0.9773028 | 0.92078067 |
| *Ifih1* | *Rnf213* | 0.709 | 0.993601198 | 0.959415625 |
| *Ifih1* | *Rtp4* | 0.823 | 0.964107129 | 0.906449676 |
| *Ifih1* | *Stat1* | 0.864 | 0.976268651 | 0.986691393 |
| *Ifih1* | *Trim21* | 0.743 | 0.901915762 | 0.979424315 |
| *Ifih1* | *Usp18* | 0.829 | 0.971576856 | 0.936207241 |
| *Ifit2* | *Ifit3* | 0.974 | 0.987008377 | 0.946689027 |
| *Ifit2* | *Igtp* | 0.734 | 0.94137058 | 0.975315452 |
| *Ifit2* | *Irf7* | 0.861 | 0.833266171 | 0.956134684 |
| *Ifit2* | *Isg20* | 0.712 | 0.747407181 | 0.910462441 |
| *Ifit2* | *Mx1* | 0.94 | 0.982095844 | 0.981204772 |
| *Ifit2* | *Oas1a* | 0.733 | 0.90625766 | 0.976363308 |
| *Ifit2* | *Oas2* | 0.882 | 0.907155963 | 0.915679002 |
| *Ifit2* | *Oas3* | 0.805 | 0.980697312 | 0.983269744 |
| *Ifit2* | *Oasl* | 0.91 | 0.917931232 | 0.961862584 |
| *Ifit2* | *Rtp4* | 0.719 | 0.944690095 | 0.951423183 |
| *Ifit2* | *Stat1* | 0.849 | 0.946209295 | 0.963890077 |
| *Ifit2* | *Usp18* | 0.853 | 0.971132859 | 0.971548932 |
| *Ifit3* | *Irf7* | 0.959 | 0.881695954 | 0.93537977 |
| *Ifit3* | *Irf9* | 0.844 | 0.95722581 | 0.797218475 |
| *Ifit3* | *Lgals3bp* | 0.825 | 0.85071944 | 0.789124094 |
| *Ifit3* | *Mx1* | 0.954 | 0.995572115 | 0.962983219 |
| *Ifit3* | *Oas1a* | 0.924 | 0.941605475 | 0.965549314 |
| *Ifit3* | *Oas2* | 0.889 | 0.954762843 | 0.873129894 |
| *Ifit3* | *Oas3* | 0.845 | 0.994928174 | 0.912442726 |
| *Ifit3* | *Oasl* | 0.976 | 0.946204426 | 0.923491344 |
| *Ifit3* | *Parp14* | 0.788 | 0.954916711 | 0.916758199 |
| *Ifit3* | *Parp9* | 0.775 | 0.974733646 | 0.969668528 |
| *Ifit3* | *Rnf213* | 0.761 | 0.987599264 | 0.904083152 |
| *Ifit3* | *Rtp4* | 0.856 | 0.977944518 | 0.985661624 |
| *Ifit3* | *Stat1* | 0.86 | 0.981809468 | 0.859051593 |
| *Ifit3* | *Usp18* | 0.945 | 0.984250456 | 0.9811365 |
| *Igtp* | *Irf9* | 0.725 | 0.883965506 | 0.957924712 |
| *Igtp* | *Irgm* | 0.86 | 0.978373319 | 0.991235333 |
| *Igtp* | *Parp14* | 0.842 | 0.857714414 | 0.995153749 |
| *Igtp* | *Parp9* | 0.724 | 0.894363819 | 0.954166007 |
| *Igtp* | *Stat1* | 0.827 | 0.946474428 | 0.973434894 |
| *Il12rb2* | *Stat1* | 0.777 | 0.879079399 | 0.826189855 |
| *Il18* | *Stat1* | 0.743 | 0.886878482 | 0.790920487 |
| *Il18* | *Tlr7* | 0.71 | 0.965438672 | 0.853952113 |
| *Irf7* | *Irf9* | 0.801 | 0.835293899 | 0.911777618 |
| *Irf7* | *Isg20* | 0.716 | 0.918906404 | 0.886849837 |
| *Irf7* | *Lgals3bp* | 0.89 | 0.989835832 | 0.936066133 |
| *Irf7* | *Mx1* | 0.983 | 0.865786522 | 0.97877068 |
| *Irf7* | *Oas1a* | 0.968 | 0.988439407 | 0.984441638 |
| *Irf7* | *Oas2* | 0.851 | 0.876956477 | 0.984433142 |
| *Irf7* | *Oas3* | 0.702 | 0.885334785 | 0.977871828 |
| *Irf7* | *Oasl* | 0.968 | 0.9822825 | 0.996934807 |
| *Irf7* | *Parp14* | 0.726 | 0.768582435 | 0.974996088 |
| *Irf7* | *Parp9* | 0.778 | 0.820282438 | 0.967868225 |
| *Irf7* | *RT1-S3* | 0.808 | 0.965938772 | 0.966517032 |
| *Irf7* | *Rtp4* | 0.776 | 0.951811401 | 0.974329442 |
| *Irf7* | *Stat1* | 0.951 | 0.896386535 | 0.910154703 |
| *Irf7* | *Tlr7* | 0.858 | 0.772827335 | 0.843110866 |
| *Irf7* | *Usp18* | 0.954 | 0.939780002 | 0.980751278 |
| *Irf7* | *Zbp1* | 0.944 | 0.891920028 | 0.779800339 |
| *Irf9* | *Mx1* | 0.916 | 0.971783416 | 0.900122862 |
| *Irf9* | *Oas1a* | 0.743 | 0.891124773 | 0.894913359 |
| *Irf9* | *Rtp4* | 0.701 | 0.953438816 | 0.837197515 |
| *Irf9* | *Stat1* | 0.999 | 0.983545159 | 0.961339993 |
| *Irf9* | *Usp18* | 0.869 | 0.917879998 | 0.87299104 |
| *Irgm* | *Parp14* | 0.868 | 0.931152059 | 0.991246315 |
| *Irgm* | *Parp9* | 0.863 | 0.961559399 | 0.971697874 |
| *Irgm* | *Stat1* | 0.802 | 0.9893165 | 0.956252915 |
| *Irgm* | *Usp18* | 0.708 | 0.98875492 | 0.978146042 |
| *Isg15* | *Mx2* | 0.941 | 0.833486009 | 0.93111325 |
| *Isg15* | *Oasl2* | 0.854 | 0.754035052 | 0.979672154 |
| *Isg15* | *Uba7* | 0.821 | 0.827532532 | 0.741737614 |
| *Isg15* | *Ube2l6* | 0.971 | 0.979642542 | 0.929898958 |
| *Isg20* | *Oasl* | 0.763 | 0.910226443 | 0.905951607 |
| *Kif11* | *Kif22* | 0.857 | 0.988530851 | -0.754327376 |
| *Kif11* | *LOC100359539* | 0.79 | 0.934225735 | 0.809890792 |
| *Kif11* | *Mcm5* | 0.814 | 0.971409291 | 0.790972404 |
| *Kif11* | *Ncaph* | 0.831 | 0.983323949 | 0.873013254 |
| *Kif11* | *Ndc80* | 0.968 | 0.948589733 | 0.903672113 |
| *Kif11* | *Rrm2* | 0.859 | 0.932401035 | 0.952557255 |
| *Kif11* | *Uhrf1* | 0.709 | 0.970464438 | 0.935545573 |
| *Kif22* | *Mcm5* | 0.709 | 0.974814338 | -0.845115072 |
| *Kif22* | *Ndc80* | 0.806 | 0.937979805 | -0.812163368 |
| *Lgals3bp* | *Lgals9* | 0.716 | 0.996088644 | 0.9827827 |
| *Lgals3bp* | *Parp14* | 0.701 | 0.704817079 | 0.949298886 |
| *Lgals3bp* | *Usp18* | 0.73 | 0.924597473 | 0.866055 |
| *LOC100359539* | *Mcm5* | 0.71 | 0.961110107 | 0.893437297 |
| *LOC100362339* | *LOC100362751* | 0.721 | 0.942350225 | -0.881673739 |
| *LOC100362339* | *Rplp2* | 0.721 | 0.823010135 | -0.880948757 |
| *LOC100362751* | *Rps17* | 0.702 | 0.932191643 | -0.794328776 |
| *Mcm5* | *Ncaph* | 0.86 | 0.973288625 | 0.865872279 |
| *Mcm5* | *Ndc80* | 0.77 | 0.926967415 | 0.756135129 |
| *Mcm5* | *Rrm2* | 0.8 | 0.955178163 | 0.862803493 |
| *Mcm5* | *Uhrf1* | 0.897 | 0.977168223 | 0.883172379 |
| *Mlkl* | *Zbp1* | 0.717 | 0.994986242 | 0.748950797 |
| *Mrpl33* | *RGD1564606* | 0.742 | 0.947175809 | 0.845535691 |
| *Mx1* | *Oas1a* | 0.959 | 0.928829871 | 0.998750147 |
| *Mx1* | *Oas2* | 0.804 | 0.968693266 | 0.930920713 |
| *Mx1* | *Oas3* | 0.769 | 0.992396645 | 0.979753917 |
| *Mx1* | *Oasl* | 0.94 | 0.928957483 | 0.974093786 |
| *Mx1* | *Parp14* | 0.748 | 0.972577179 | 0.972643995 |
| *Mx1* | *Parp9* | 0.824 | 0.985343522 | 0.95434471 |
| *Mx1* | *Rtp4* | 0.768 | 0.975739324 | 0.977587937 |
| *Mx1* | *Stat1* | 0.905 | 0.983869674 | 0.929148918 |
| *Mx1* | *Usp18* | 0.976 | 0.97345936 | 0.994409089 |
| *Mx2* | *Oas1f* | 0.821 | 0.945426883 | 0.825847801 |
| *Mx2* | *Oasl2* | 0.845 | 0.986775556 | 0.984972286 |
| *Ncaph* | *Ndc80* | 0.862 | 0.950176437 | 0.826355692 |
| *Ndc80* | *Rrm2* | 0.77 | 0.95850531 | 0.813310508 |
| *Ndc80* | *Uhrf1* | 0.72 | 0.92392152 | 0.869476046 |
| *Oas1a* | *Oasl* | 0.708 | 0.997488878 | 0.978432099 |
| *Oas1a* | *Usp18* | 0.954 | 0.979694556 | 0.99679503 |
| *Oas2* | *Parp14* | 0.783 | 0.960547364 | 0.956766035 |
| *Oas2* | *Usp18* | 0.734 | 0.931250506 | 0.933304228 |
| *Oasl* | *Rtp4* | 0.751 | 0.979066872 | 0.960837596 |
| *Oasl* | *Usp18* | 0.934 | 0.984673261 | 0.971671137 |
| *Parp12* | *Parp14* | 0.71 | 0.951952727 | 0.99330218 |
| *Parp12* | *Parp9* | 0.817 | 0.974511098 | 0.94757536 |
| *Parp12* | *Usp18* | 0.709 | 0.986473889 | 0.972793002 |
| *Parp12* | *Znfx1* | 0.732 | 0.992415944 | 0.758421126 |
| *Parp14* | *Parp9* | 0.966 | 0.990581001 | 0.95681379 |
| *Parp14* | *Rnf213* | 0.794 | 0.985497404 | 0.988941697 |
| *Parp14* | *Rtp4* | 0.742 | 0.921088344 | 0.94265486 |
| *Parp14* | *Stat1* | 0.831 | 0.965190287 | 0.969816878 |
| *Parp14* | *Usp18* | 0.824 | 0.900390664 | 0.962086505 |
| *Parp9* | *Rtp4* | 0.742 | 0.952343571 | 0.983757531 |
| *Parp9* | *Stat1* | 0.855 | 0.984947457 | 0.890080343 |
| *Parp9* | *Usp18* | 0.824 | 0.931305381 | 0.973959714 |
| *Pml* | *Sp100* | 0.954 | 0.988999066 | 0.920107458 |
| *Psme1* | *Psme2* | 0.979 | 0.979266966 | 0.735172904 |
| *Rnf213* | *Rtp4* | 0.785 | 0.955726334 | 0.94837325 |
| *Rnf213* | *Usp18* | 0.808 | 0.954482422 | 0.960123094 |
| *RT1-S3* | *Tapbp* | 0.798 | 0.972923813 | 0.706556606 |
| *Rtp4* | *Usp18* | 0.856 | 0.987613747 | 0.994047843 |
| *Stat1* | *Usp18* | 0.872 | 0.96321677 | 0.903896469 |

**Table S5 The antibodies used for immunohistochemistry staining, Immunofluorescence analysis and western blotting.**

| **Antibody** | **Cat No.** | **Manufacturers** | **Host** | **Description** | **Specificity** | **Molecular Weight** | **Dilution** |
| --- | --- | --- | --- | --- | --- | --- | --- |
| p-RIPK1 | AF7088 | Affinity | Rabbit | Rabbit polyclonal antibody to Phospho-RIPK1 (Tyr384) | Human, Mouse, Rat | 76kDa | 1/100 |
| p-RIPK3 | AP1257 | ABclonal | Rabbit | Phospho-RIP3-S227 Rabbit pAb (AP1257) | Human, Mouse, Rat | 57kDa | 1/100 |
| p-MLKL | AF7420 | Affinity | Rabbit | Rabbit polyclonal antibody to Phospho-MLKL (Ser358) | Human, Mouse, Rat | 54kDa | 1/100 |
| p-STAT1 | AP0453 | ABclonal | Rabbit | Phospho-STAT1-S727 Rabbit pAb (AP0453) | Human, Mouse, Rat | 87kDa | 1/200 |
| FITC | 71-1900 | Invitrogen | Goat | Goat anti-Rabbit IgG (H+L) Cross-Adsorbed Secondary Antibody, FITC | Rabbit | - | 2 µg/mL |
| ZBP1 | A13899 | ABclonal | Rabbit | ZBP1 Rabbit pAb (A13899) | Human, Mouse, Rat | 58kDa | 1/5000 |
| p-PKR | orb6786 | Biorbyt | Rabbit | PKR (phospho-Thr446/451) antibody | Human, Mouse, Rat | 62kDa | 1/1000 |
| PKR | BF8222 | Affinity | Mouse | Mouse monoclonal antibody to PKR | Mouse, Rat | 62kDa | 1/1000 |
